# Humin oxidation drives microbial dehalogenation in oligotrophic environments

**DOI:** 10.1093/ismejo/wraf207

**Published:** 2025-09-17

**Authors:** Zimeng Zhang, Xing Liu, Zhiling Li, Xueqi Chen, Yunxia Zu, Shih-Hsin Ho, Bin Liang, Shungui Zhou, Aijie Wang

**Affiliations:** State Key Laboratory of Urban-rural Water Resource and Environment, School of Environment, Harbin Institute of Technology, Harbin, Heilongjiang 150090, China; Fujian Provincial Key Laboratory of Soil Environmental Health and Regulation, College of Resources and Environment, Fujian Agriculture and Forestry University, Fuzhou, Fujian 350002, China; State Key Laboratory of Urban-rural Water Resource and Environment, School of Environment, Harbin Institute of Technology, Harbin, Heilongjiang 150090, China; State Key Laboratory of Urban-rural Water Resource and Environment, School of Environment, Harbin Institute of Technology, Harbin, Heilongjiang 150090, China; State Key Laboratory of Urban-rural Water Resource and Environment, School of Environment, Harbin Institute of Technology, Harbin, Heilongjiang 150090, China; State Key Laboratory of Urban-rural Water Resource and Environment, School of Environment, Harbin Institute of Technology, Harbin, Heilongjiang 150090, China; School of Eco-Environment, Harbin Institute of Technology, Shenzhen, Guangdong 518055, China; Fujian Provincial Key Laboratory of Soil Environmental Health and Regulation, College of Resources and Environment, Fujian Agriculture and Forestry University, Fuzhou, Fujian 350002, China; State Key Laboratory of Urban-rural Water Resource and Environment, School of Environment, Harbin Institute of Technology, Harbin, Heilongjiang 150090, China; School of Eco-Environment, Harbin Institute of Technology, Shenzhen, Guangdong 518055, China

**Keywords:** humin oxidation, organohalide biotransformation, oligotrophic survival, multiheme cytochrome

## Abstract

Energy acquisition presents a fundamental constraint for microbial survival in oligotrophic environments. Although heterotrophic organohalide-respiring bacteria (OHRB) are known to perform reductive dehalogenation in organohalide-contaminated oligotrophic ecosystems, their energy metabolism remains poorly understood. Here, we report that *Pseudomonas* sp. CP-1, an organohalide-respiring bacterium, can directly oxidize humin from diverse oligotrophic aquifers to drive organohalide respiration. Spectroscopy, electrochemistry, and metabolic profiling demonstrated that electrons stored in phenolic hydroxyl and amino groups of humin were utilized by strain CP-1 for organohalide respiration. Mutational and chemical inhibition studies identified an extracellular electron uptake pathway involving a multiheme cytochrome EeuP, which transfers extracellular electrons into the organohalide-respiratory chain, thereby coupling humin oxidation with reductive dehalogenation. Phylogenetic analyses revealed the widespread distribution of EeuP homologs across environmental bacterial taxa, implying a broader ecological relevance. This discovery sheds light on the hidden world of subsurface microbiology, with implications for understanding microbial energy metabolism in the energy-scarce environments.

## Introduction

Energy acquisition is essential for microbial survival, growth, and ecological functioning [1]. The primary natural energy sources are chemical energy stored in chemical bonds and solar radiation. Microorganisms have evolved diverse strategies to exploit these sources, including phototrophy (light harvesting in euphotic zones) [2], organotrophy (organic matter decomposition) [3], and lithotrophy (inorganic substrate oxidation) [4]. Recently identified electrotrophy enables direct electrical energy use from environmental sources [5, 6]. This metabolic versatility underpins microbial persistence across diverse habitats and drives global biogeochemical cycles, including carbon sequestration, nutrient turnover, and pollutant degradation [7]. Such adaptations are critical in oligotrophic environments (e.g. groundwater, marine sediments, peatlands), which cover ~50% of Earth’s surface yet impose severe energy constraints [8, 9]. Here, microorganisms employ specialized survival strategies including enhanced nutrient acquisition, metabolic streamlining, and alternative energy exploitation (e.g. trace aqueous ammonium and atmospheric trace gases) [10–13].

Microbial reductive dehalogenation commonly occurs in organohalide-contaminated oligotrophic groundwater and represents another typical case of microbial oligotrophy [14–16]. Reductive dehalogenation is a respiratory process in which organohalide-respiring bacteria (OHRB) transfer electrons to remove halogens, thereby conserving energy [17]. OHRB are generally heterotrophic. Although their respiratory biochemistry has been widely studied in nutrient-rich systems [18–20], a fundamental question remains unresolved: how do they sustain energy metabolism under oligotrophic conditions where conventional organic substrates are extremely limited? Current paradigms suggest that OHRB primarily depend on electron donors supplied by fermentative partners or exogenous amendments [21, 22]. However, such mechanisms may be thermodynamically unsustainable in deeply oligotrophic zones lacking labile carbon.

Humic substances, complex heterogeneous organic polymers formed by the decay of biomass, represent the largest pool of reduced carbon in oligotrophic subsurface ecosystems [23–25]. Within this pool, humin (the insoluble, macromolecular fraction that remains after extraction of humic and fulvic acids) is particularly dominant due to its recalcitrance and near-ubiquity [26]. Moreover, humin contains abundant redox-active moieties, such as phenolic hydroxyl and quinone groups, which confer significant electron-donating capacity (EDC) [26, 27]. Although humin is known to act as an electron shuttle for microbial reductive dehalogenation [27, 28], the direct biological oxidation of insoluble humin particles as a primary respiratory electron source to conserve energy remains unexplored—despite its potential to support the sustainable metabolism of OHRB in organohalide-contaminated oligotrophic systems.

In this study, we first investigated microbial reductive dechlorination in organohalide-contaminated aquifer material microcosms. We investigated the energy acquisition of *Pseudomonas* sp. CP-1, a facultative OHRB previously isolated from oligotrophic groundwater [29]. Our results show that strain CP-1 recruits an extracellular electron uptake (EEU) pathway including a multiheme cytochrome EeuP to couple humin oxidation with reductive dechlorination, thereby achieving organohalide respiration. Phylogenetic surveys further indicate the prevalence of EeuP homologs across diverse microbial taxa, suggesting widespread ecological significance. Our study uncovers a previously hidden mechanism of microbial energy harvesting in the subsurface, with implications for bioremediation and our understanding of life in oligotrophic environments.

## Materials and methods

### Field sampling, humin extraction, and characterization

Aquifer materials were collected from the saturated zone (20 cm below the groundwater table) at five 2,4,6-trichlorophenol (TCP)-contaminated sites across China. Redox potential was measured *in situ* using a calibrated portable meter (SX751, Sanxin, China). Fresh samples were wet-sieved through a 2 mm stainless steel mesh to remove coarse debris, wrapped in aluminum foil, sealed in sterile plastic bags purged with ultrapure nitrogen, and transported on ice. Upon receipt, samples were stored at −20°C under a nitrogen atmosphere.

Fulvic acid, humic acid, and humin were extracted from 100 g of samples following modified International Humic Substances Society protocols [30] (Supplementary Text S1). Extracted humin was oxidized by 30% (m/v) H_2_O_2_ (6 h, 25 ± 2°C) or reduced by 0.1 M NaBH_4_ (15 h) under nitrogen-purged anaerobic conditions, yielding oxidized humin or reduced humin, respectively [28]. Functional groups were characterized by Fourier-transform infrared spectroscopy (FTIR, Nicolet iN10, Thermo, USA) using the KBr pellet method (1 mg humin with 200 mg KBr; scan range 400–4000 cm^−1^, resolution 4 cm^−1^, 64 scans), and by X-ray photoelectron spectroscopy (XPS, Axis Ultra DLD, Kratos, USA) with an Al Kα source (12 kV, 6 mA; survey scan 100 eV, high-resolution scan 30 eV).

The EDC of humin was quantified via mediated electrochemical oxidation, as previously described [31]. Briefly, a three-electrode system (glassy carbon working electrode, platinum counter electrode, saturated calomel reference electrode) was used with 0.1 M phosphate buffer (purged with ultrapure nitrogen) as the electrolyte. 2,2′-Azino-bis(3-ethylbenzthiazoline-6-sulfonic acid) (ABTS) served as the mediator. Humin was applied to the glassy carbon electrode at +0.61 V and EDC was calculated by integrating the area under the oxidative current [31].

### Strain culture and microcosm experiments

Anaerobic microcosms were established in sealed serum bottles containing 5 g of aquifer material, 100 ml of mineral salts medium (MSM), and 100 μm TCP. Pure culture experiments were performed in 100 ml of MSM with 100 μm TCP, strain CP-1 (cell density of 9.4 ± 2.3 × 10^6^ cells ml ^−1^), and either 500 mg (dry weight) of humin or 5 mm acetate. Before inoculation, strain CP-1 cells were washed twice by centrifugation (6000 g, 10 min, 4°C) and resuspended in fresh MSM to remove residual substrates. All cultures were incubated at 30°C in the dark. Details on MSM composition are described in Text S2.

### Characterization of extracellular electron uptake

Extracellular electron uptake was characterized in a sealed two-chamber bioelectrochemical system (BES, total volume of 95 ml, effective volume of 80 ml) separated by a Nafion117 proton exchange membrane [32]. Graphite fiber brushes (3 cm diameter × 3 cm length) served as both working and counter electrodes, and a saturated calomel electrode (SCE) was used as reference. The catholyte consisted of MSM containing 100 μm TCP, and the anolyte contained 50 mm potassium ferricyanide in phosphate buffer (pH 7.0). The cathode was poised at −0.5 V using a CHI660 potentiostat (Chenhua, China) until a stable current was achieved, after which strain CP-1 was inoculated. The current was recorded simultaneously.

In situ electrochemical FTIR spectroscopy was performed using a Nicolet 6700 spectrometer (Thermo Fisher, USA) equipped with a spectroelectrochemical cell featuring glassy carbon as the working electrode, platinum as the counter electrode, and SCE as the reference electrode [33]. Cathodic biofilm samples from the BES were deposited on polished glassy carbon electrodes. Stepwise potentials from −0.7 V to +0.1 V (in 0.2 V increments) were applied on the working electrode with spectra collected at each potential after equilibration and referenced to the spectrum obtained at the open circuit potential.

### Proteomic and protein analysis

Proteomics analysis was conducted with ~10^9^ cells using tandem mass tag (TMT) labeling and high-resolution mass spectrometry (Orbitrap Exploris 480, Thermo Fisher Scientific, USA). Mass spectra were searched against a protein database derived from the genome of strain CP-1 (GeneBank: CP135173.1) using the SEQUEST HT algorithm in Proteome Discoverer (v2.4), with peptide-spectrum matches filtered at a false discovery rate of 1% [34]. Putative cytochrome *c* proteins were identified by hidden Markov model (HMM) search (PF00034, PF13442, PF21342; e-value <1e^−5^, alignment >50%) for CXXCH motif [35, 36]. Cytochromes were regarded as membrane-anchored or extracellularly secreted only if possessing either transmembrane helices (TMHMM-2.0) or signal peptides (SignalP-6.0) [37, 38].

### Quantitative PCR

Quantitative PCR was performed on a real-time PCR detection system (CFX96, Bio-Rad, USA) following the MIQE (Minimum Information for Publication of Quantitative Real-Time PCR Experiments) guidelines and EMMI (Environmental Microbiology Minimum Information) recommendations [39, 40]. For absolute quantification, standards were made from cloned *fixA*, *fixB,* and *cprA* genes amplified from the strain CP-1 genome. Protocol details and primers are provided in Text S3 and Table S1, respectively.

### Mutant construction

The *eeuP* gene (gene4129, WP_003082412.1) was knocked out via double homologous recombination, as previously reported [41]. A gentamicin resistance cassette, along with ~800 bp upstream and downstream homologous arms of *eeuP*, was assembled into the suicide plasmid pCVD442 to construct pCVD442-Δ*eeuP*::Gm. This recombinant plasmid was delivered to strain CP-1 via conjugation with *Escherichia coli* β2155. Transconjugants were selected on gentamicin plates. Positive colonies were cultured in Luria-Bertani (LB) medium and streaked on LB plates containing 10% (w/v) sucrose for counterselection. The final mutant was verified by PCR and designated as Δ*eeuP*. Protocol details and primers are provided in Text S4 and Table S1, respectively.

### Gene retrieval and phylogenetic tree construction

EeuP homologs were identified by PSI-BLAST (5 iterations, e-value <1e^−6^) against the nonredundant protein database [42]. To reduce redundancy and ensure sequence diversity, retrieved sequences were clustered using MMseqs2 (50% identity, >80% coverage) and sequences shorter than 100 or longer than 400 amino acids were excluded. Multiple sequence alignment was performed with MUSCLE (v5.1), and positions with >20% gaps were trimmed.

63 OHRB genomes were retrieved from the NCBI GenBank database (Supplementary Data S1). A genome was considered to possess EeuP homologs only when both of the following criteria were satisfied [43, 44]: (i) HMM search with the EeuP domain (PF21342; e-value < 1e^−5^, coverage > 50%); (ii) BLASTP against the reference EeuP (e-value < 1e^−5^, identity > 30%, bit score > 30).

### Analytical methods

Freeze-dried aquifer materials were analyzed for total organic carbon (TOC) using a multi-N/C 3100 analyzer (Analytik Jena) with high-temperature catalytic combustion and nondispersive infrared detection. TCP, 2,4-dichlorophenol (2,4-DCP) and 4-chlorophenol (4-CP) were quantified by ultra-high-performance liquid chromatography (ICS2100, Waters, USA) equipped with a reversed-phase C18 column (50 × 2.1 mm) and a photodiode array detector. The analysis was performed with a methanol–water gradient elution (60:40–10:90, v/v) at a flow rate of 0.1 ml min^−1^ [29]. Hydrogen was quantified using a GC7890 gas chromatograph (Agilent, CA) equipped with a TDX-01 column (2 m × 2 mm) and a thermal conductivity detector, with a detection limit of 10 ppm. TCP dechlorination rates (*k*) were calculated using a previously reported pseudo-first-order model [29]. The detail is provided in Text S5.

## Results

### Microbial reductive dehalogenation in oligotrophic environments.

**Figure 1:**
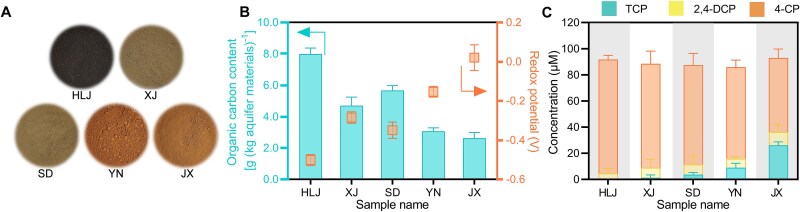
Trichlorophenol reductive dehalogenation in five different aquifer materials. (A) Appearance of five aquifer materials. Samples were collected from five Chinese provinces: Heilongjiang (HLJ), Xinjiang (XJ), Shandong (SD), Yunnan (YN), and Jiangxi (JX). (B) Organic carbon contents and *in situ* redox potentials; bars (left) and boxes (right) indicate organic carbon content and redox potential, respectively. (C) Reduction of 2,4,6-trichlorophenol (TCP) in microcosms containing aquifer materials. TCP underwent stepwise reductive dechlorination under the action of the microbiome, with 2,4-dichlorophenol (2,4-DCP) as an intermediate metabolite and 4-chlorophenol (4-CP) as the final metabolite. Data represent the mean of three biological replicates. Error bars indicate standard deviations.

Groundwater ecosystems are typically oligotrophic, with limited organic carbon availability [45]. Previous studies have indicated that heterotrophic OHRB can perform organohalide respiration in organohalide-contaminated groundwater ecosystems [46]. We collected aquifer materials from five 2,4,6-trichlorophenol (TCP)-contaminated groundwater ecosystems across China. These samples exhibited distinct colors: dark-brown (Heilongjiang), yellow (Shandong and Xinjiang), and reddish–brown (Jiangxi and Yunnan) (Fig. 1A). In addition, the samples displayed varying redox potentials. The dark-brown sample had a redox potential of ~−0.50 V, which was lower than that of the yellow sample, whereas the reddish–brown sample had the highest redox potential (Fig. 1B). The color differences likely result from variations in humic substance content, and the redox potential disparities indicate differences in organic carbon content [47, 48]. However, the TOC content in all samples was below 1% of dry weight (Fig. 1B), confirming oligotrophic conditions in these ecosystems [49]. To identify the possibility of microbial dehalogenation in those oligotrophic environments, we monitored the biotransformation of TCP in microcosms constructed with aquifer materials from the five ecosystems. As indicated in Fig. 1C, TCP was reduced to 2,4-dichlorophenol (2,4-DCP) and 4-chlorophenol (4-CP) in all samples, with reduction ratio varying from 62.8 ± 3.3% to 100.0 ± 7.8% depending on the aquifer material source. By contrast, TCP remained intact in autoclaved aquifer materials (Fig. S1A). In particular, dehalogenase gene (*cprA*), the biomarker of microbial TCP reduction [29, 50], was actively expressed in all nonautoclaved aquifer materials (Fig. S1B). These results suggest that the TCP dehalogenation was biotic rather than abiotic, and was facilitated by microorganisms. However, considering the oligotrophic nature of aquifers, the electron source for microbial TCP reduction was unclear.

### Humin can be an electron donor to support reductive dehalogenation.

**Figure 2:**
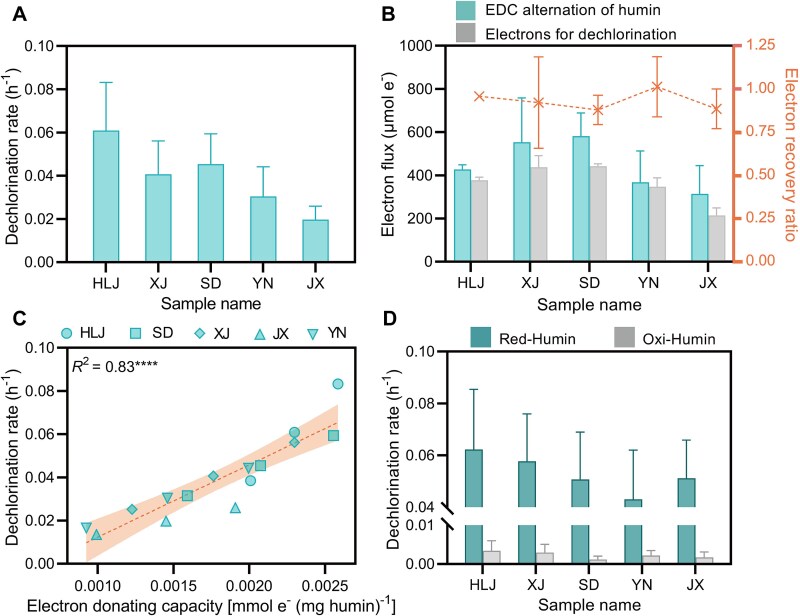
Correlation of electron-donating capacity of humin with dechlorination rate. (A) Dechlorination rate of trichlorophenol (TCP), determined by fitting TCP consumption data to a pseudo-first-order kinetic model. Humins extracted from aquifer materials in Heilongjiang (HLJ), Xinjiang (XJ), Shandong (SD), Yunnan (YN), and Jiangxi (JX) were tested in microcosms. (B) Electron flux and electron recovery calculation. The change in electron-donating capacity (EDC) of humin was determined by measuring the EDC of humin before and after dechlorination. The electrons for dechlorination were determined based on the electron consumption required to produce the end products: 2 mol of electrons are required to generate 1 mol of 2,4-dichlorophenol, and 4 mol of electrons are required for 1 mol of 4-chlorophenol. Electron recovery was calculated as the ratio of electrons used for dichlorination to the change in humin’s EDC. (C) Positive correlation between EDC of humin and dehalogenation rate, determined by Pearson correlation analysis. (D) Trichlorophenol dechlorination in response to oxidized humin (Oxi-Humin) and reduced humin (Red-Humin), which were produced by pretreating humin with H_2_O_2_ and NaBH_4_, respectively. The data represent the mean of triplicate samples, and error bars indicate standard deviations.

Previous studies indicated that humic substances can provide electrons to drive microbial reductive respiration [24, 27, 51–53], suggesting their potential role as electron donors for microbial reductive dehalogenation in groundwater ecosystems. Humic substances consist of fulvic acid, humic acid, and humin. We isolated these three components from each aquifer material. In previous studies, we isolated an organohalide-respiring bacterium, *Pseudomonas* sp. CP-1, from the TCP dehalogenation aquifer [29]. Strain CP-1 could not use H_2_ as an electron donor (Fig. S2), but was capable of performing EEU to accept electrons from the electrode for organohalide respiration (Fig. S3). Here, the ability of humic substances to serve as electron donors to support TCP reduction by strain CP-1 was tested. Humin was the most abundant fraction of the humic substances (>50%) and contributed to nearly all the TCP reduction, whereas humic acid or fulvic acid alone had a negligible effect on the dechlorination (Fig. S4). In particular, the dechlorination rate varied from 0.019 ± 0.0050 h^−1^ to 0.061 ± 0.018 h^−1^, with a descending order for the humins from Heilongjiang, Shandong, Xinjiang, Jiangxi, and Yunnan Provinces (Fig. 2A and S5).

Previous studies indicated that humin is redox-active with the capability to serve as an electron donor to power reductive dehalogenation [27]. Given that humin is a macromolecular polymer primarily composed of aromatic compounds, aliphatic species, carbohydrates, nitrogen-containing compounds, and lignin [54], we examined the possibility that humin metabolism supports dechlorination of strain CP-1. We monitored the structural integrity of humin during the reduction of TCP *via* FTIR and XPS, which can be used to identify the structure of humin and quantify functional groups, respectively. FTIR spectra showed that both the intensity and width of peaks arising from aromatic C–H stretching at 779 cm^−1^ and CH_2_ stretching at 2851 and 2991 cm^−1^ remained unchanged during dechlorination (Fig. S6), indicating the preservation of the carbon skeleton of humin. Therefore, the oxidation of humin organic carbon is apparently not responsible for the reduction of TCP. By contrast, XPS spectra revealed an increase in the relative content of oxidized groups, such as quinone-related groups (C=O and O–C=O, from 14.71% to 17.33%) and a decrease in reduced groups, such as amino groups (from 96.0% to 79.5%) and phenolic hydroxyl groups (from 33.6% to 24.7%) in the humin during the reduction of TCP (Fig. S7). The alterations in these functional groups indicate humin oxidation and suggest an EDC of humin.

We measured the EDC of different humins at the beginning and the end of the dechlorination and calculated the corresponding electron loss (Fig. S8). We also estimated the electrons invested for dechlorination by calculating the reduction of TCP (Fig. S5). A stoichiometric balance with a ratio of 0.879 ± 0.069–1.013 ± 0.142 between electron loss and electrons used for TCP reduction was observed (Fig. 2B). In particular, the differences in dechlorination rates among humins showed a positive correlation with their EDC (*R*^2^ = 0.83, *P* < .0001, Fig. 2C). We also chemically modulated the EDC of humins and subsequently monitored their effects on the dechlorination by strain CP-1 (Fig. S5). As indicated in Fig. 2D, reduced humins (electronically charged) contributed to higher dechlorination rates (0.39–0.98 times higher than that of untreated humin), whereas oxidized humins (electronically discharged) barely facilitated TCP reduction. These results collectively demonstrate that humin oxidation directly drives dechlorination by strain CP-1.

### Strain CP-1 uses a cytochrome-based extracellular electron uptake pathway to oxidize humin.

**Figure 3:**
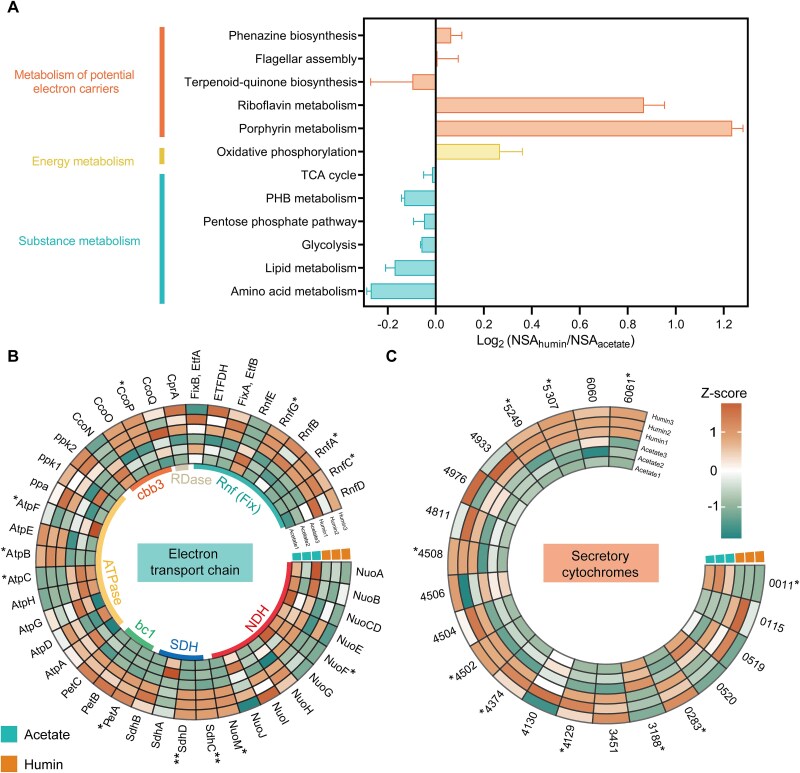
Comparative proteome profiling reveals distinct expression patterns in humin-oxidizing *versus* acetate-fed cells of *Pseudomonas* sp. CP-1. (A) The relative expression of proteins involved in various pathways in strain CP-1, which are presented as a normalized score of protein activity (NSA). Differentially expressed (^*^P < .05; ^**^P < .01) (B) putative proteins responsible for electron transport and (C) secretory cytochromes are shown in heatmaps. The three biological replicates are displayed side-by-side. *Z*-scores were calculated to normalize protein abundance. NDH: NADH dehydrogenase; SDH: succinate dehydrogenase; bc_1_: cytochrome *bc*_1_ complex; ATPase: ATP synthase; cbb3: Cytochrome *cbb*_3_ terminal oxidase; RDase: reductive dehalogenase; Rnf: Rnf complex, Etf (Fix): electron transfer flavoprotein.

To understand the coupling of humin oxidation with the organohalide respiration of strain CP-1, we compared proteomes of cells raised with humin against cells grown on acetate. Among 2218 identified proteins, 358 were upregulated and 318 were downregulated in humin-oxidizing cells (Data S2). Kyoto Encyclopedia of Genes and Genomes analysis revealed that the differentially expressed proteins were enriched in pathways related to substance metabolism, energy conversion, and cofactor synthesis (Fig. 3A). Metabolic pathways responsible for cellular metabolism [e.g. fatty acid degradation and the tricarboxylic acid cycle (TCA cycle)] were downregulated by 1.1%–17.1% (Fig. 3A), which was consistent with the observation that reductive dechlorination by strain CP-1 did not result from metabolic humin degradation. By contrast, the oxidative phosphorylation pathway was upregulated by 120.5% in humin-oxidizing cells compared to acetate-fed cells (Fig. 3A), indicating the involvement of an electron transport chain (ETC) in the humin oxidation. In particular, succinate dehydrogenase (SDH) and electron transport-associated enzymes [such as the ubiquinol: cytochrome *c* oxidoreductase complex (cytochrome *bc*_1_ complex), *cbb*_3_-type cytochrome *c* oxidase, H^+^/Na^+^-translocating ferredoxin: NAD^+^ reductase complex (Rnf), and electron-transferring flavoprotein complex (Etf)] exhibited significant upregulation (Fig. 3B, Data S3).

**Figure 4:**
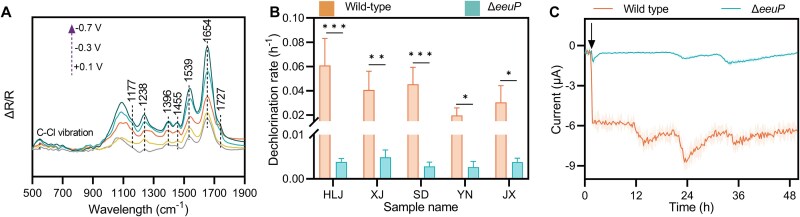
Multiheme cytochrome EeuP facilitates extracellular electron uptake by strain CP-1. (A) In situ electrochemical Fourier-transform infrared spectra of strain CP-1 under different applied electrode potentials. The y-axis (ΔR/R) indicates the normalized change in infrared reflectance, where ΔR corresponds to the difference in reflectance measured at a given potential relative to the reflectance at the open-circuit potential (R). (B) Trichlorophenol dechlorination rates of wild-type strain CP-1 and the Δ*eeuP* mutant when humins from different aquifers were provided as electron donors. A significant difference (*P* < .05) was observed in the dechlorination rates between the wild-type and Δ*eeuP* mutant. HLJ, XJ, SD, YN, and JX are abbreviations corresponding to the samples isolated from Heilongjiang, Xinjiang, Shandong, Yunnan, and Jiangxi Provinces, respectively. (C) The extracellular electron uptake of wild-type strain CP-1 and Δ*eeuP* mutant. The cathode was held at −0.5 V. Dark arrow indicates cell addition; equal cell numbers were inoculated. The shaded area represents one standard deviation. The results shown are means ± standard deviation for triplicate samples.

The significant upregulation of porphyrin biosynthesis pathway (Fig. 3A) suggested the involvement of cytochromes in humin oxidation because membrane-associated cytochromes have been suggested to facilitate EEU and porphyrin is a key functional component of the heme group(s) in cytochromes [55, 56]. To identify the EEU mechanism of strain CP-1, we monitored its EEU using electrochemical FTIR. A gradient potential from low (−0.7 V) to high (+0.25 V) was applied to induce the EEU, and the corresponding FTIR spectra were recorded (Fig. 4A). Only spectra in the infrared region of cytochromes (1800–1100 cm^−1^) exhibited shifts or vibrations with changes in potential [33]. Specifically, the variations at 1727 cm^−1^ and 1539 cm^−1^ were attributed to the stretching vibration of carboxyl groups and the deformation of N–H bonds in the polypeptide amide II region, respectively; the variation at 1654 cm^−1^ arose from the strong stretching vibration of carbonyl groups in the polypeptide amide I region [33]. These results indicate that cytochromes undergo polarization during EEU. Moreover, inhibiting the electron transfer capability of outer membrane cytochromes via *N*-acetylmethionine treatment suppressed the EEU (Fig. S9). These results reflect that redox-active cytochromes facilitate electron transfer across outer membranes of strain CP-1.

We further compared the expression of cytochromes during humin oxidation to that during acetate oxidation. A total of 56 putative *c*-type cytochrome-encoding genes were identified in the genome of strain CP-1 (Data S4) using the signature CXXCH amino acid motif (the heme-binding site) as a query, as previously indicated [37]. Only 21 were predicted to be secretory proteins (Data S5). Of those, only six putative cytochromes (genes 2383, 4129, 4502, 4508, 5307, and 6061) were significantly upregulated (*P* < .05) in humin-oxidizing cells (Fig. 3B, Data S6). Among them, it was predicted that genes 4502 and 4508 belong to cytochrome *c* oxidase inner membrane *cbb*_3_-type subunit III; genes 2382, 5307, and 6061 encode extracellular cytochromes, only gene 4129 encodes a transmembrane cytochrome (Fig. S10), suggesting potential involvement in EEU. Gene 4129, hereafter named *eeuP*, encodes a multiheme cytochrome because it has four CXXCH motifs (Fig. S10). An *eeuP* deletion mutant (Δ*eeuP*) of strain CP-1 was constructed to verify the function of EeuP in EEU. The mutation neither impaired the cell growth nor affected dehalogenation when acetate was used as the electron donor (Table S2). However, the reduction of TCP by the Δ*eeuP* strain was only 6.2%–15.2% of that by the wild-type strain when humins from different aquifer materials were oxidized (Fig. 4B). Simultaneously, the EEU of the Δ*eeuP* mutant was decreased to 11.2 ± 0.3% of that of the wild-type strain (Fig. 4C). The incomplete inhibition of EEU suggests functional redundancy, likely attributable to promiscuous electron transfer from other membrane-associated cytochromes. These results indicate that cytochrome EeuP is a key component of the EEU pathway in strain CP-1, which directly contributes to humin oxidation.

**Figure 5:**
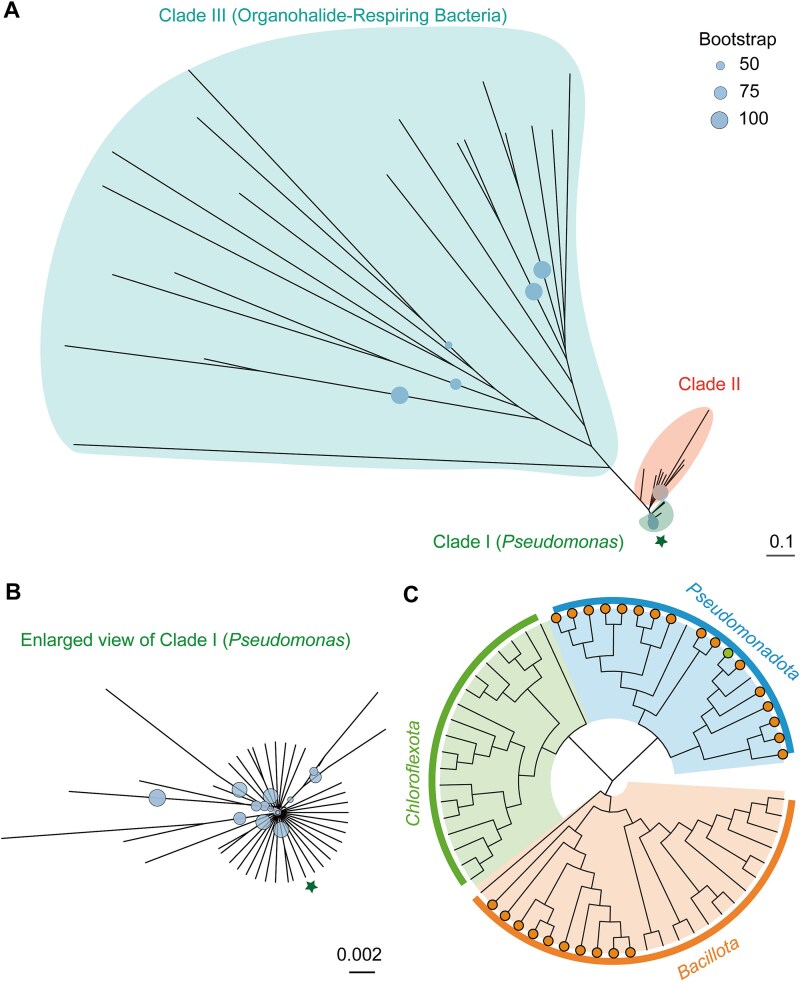
Cytochrome EeuP is widely distributed among microorganisms. (A) Maximum likelihood phylogenetic tree of EeuP homologs constructed using the Jones-Taylor-Thornoton (JTT) model with 1000 bootstrap replicates. Tree branches are grouped by clade, with major clade names labeled. The position of EeuP is marked with a star symbol. Bootstrap support values greater than 50% are indicated by circles. The scale bar represents the number of amino acid substitutions per site. (B) Enlarged view of clade I (*Pseudomonas*) from the phylogenetic tree in panel (A). (C) Cladogram of 63 organohalide-respiring bacteria based on 16S rRNA gene sequences, constructed using MUSCLE alignment and Kimura 2-parameter model. Tree branches are grouped by phylum, with major clade names labeled. Species containing EeuP homologs are indicated via filled circles at the ends of nodes.

We analyzed the phylogenetic distribution of EeuP homologs. The analysis revealed that they clustered into three major clades (Fig. 5A and B and S11). Clade I comprises *Pseudomonas* species and closely related taxa, forming a highly conserved monophyletic group; clade II is closely related to clade I and includes facultative aquatic bacteria such as *Dongia*, *Shewanella*, and *Dyella*; clade III is more distantly related to clade I and is primarily composed of canonical OHRB, but exhibiting significant phylogenetic diversity and including *Anaeromyxobacter*, *Desulfuromonas*, *Sulfurospirillum*, and *Dehalogenimonas*. In particular, most of these species showed extracellular electron transfer activities [57, 58]. This distribution suggests the conservation of EeuP in *Pseudomonas* and its potential role in contributing to the extracellular electron transfer in other species. Also, EeuP homologs were found in 26 strains among 63 fully sequenced OHRB, mainly in the phyla *Pseudomonadota* and *Bacillota* (Fig. 5C and S11). These species have been discovered in various oligotrophic dehalogenation environments [15, 59, 60]. This suggests that EeuP-mediated EEU may also be used by other OHRB to sustain reductive dehalogenation functions in energy-limited conditions.

### Coupling humin oxidation with organohalide respiration in strain CP-1.

**Figure 6:**
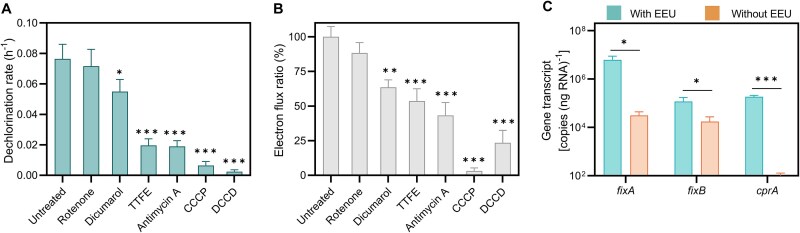
The organohalide-respiratory electron transfer chain is involved in humin oxidation-driven reductive dichlorination. (A) Dechlorination rate and (B) extracellular electron uptake. Different electron transport chain complex protein inhibitors (final concentration: 100 μm) were added when a stable cathodic current was generated. Electron consumption during microbial dechlorination was calculated by integrating the current with time and normalized to untreated cells (control). The probes and their inhibition sites were as follows: Rotenone for NADH dehydrogenase, dicumarol for ubiquinone, thenoyltrifluoroacetone (TTFE) for succinate dehydrogenase, antimycin a for the cytochrome *bc*_1_ complex, carbonyl cyanide *m*-chlorophenyl hydrazine (CCCP) for the proton motive force, and dicyclohexylcarbodiimide (DCCD) for ATP synthase. (C) Gene expression analysis. Gene transcripts of electron bifurcation complex *fixAB* and dehalogenase *cprA* were quantified. Cells grown with a polarized (with EEU) or a depolarized (without EEU) electrode as an extracellular electron source were compared. The electrode was poised at −0.5 V or open circuit potential. Data represent the average of triplicate tests and error bars indicate standard deviations. ^*^P < .05, ^**^P < .01, ^***^P < .001.

Previous studies indicated that the organohalide-respiratory ETC was composed of NADH: quinone oxidoreductase (NDH), SDH, cytochrome *bc*_1_, reductive dehalogenase (CprA), and other electron transport-associated enzymes [29]. The proteomic data above demonstrated that some of these components were upregulated during humin oxidation, suggesting that the organohalide-respiratory ETC contributes to coupling humin oxidation with organohalide reduction. To investigate how EEU couples with organohalide reduction, we inhibited the activity of each constituent in the organohalide-respiratory ETC separately using an adapted chemical-probe-based approach, and monitored the EEU and TCP reduction [37]. The addition of various inhibitors did not lead to any significant changes in cell viability (Fig. S12), demonstrating the feasibility of chemical probe treatment, as previously indicated [61]. Quinone reduction is recognized as the primary mechanism by which the cell harvests electrical energy [62]. Disrupting the quinone loop with dicumarol [63] led to a remarkable decrease (*P*<.05) in EEU and the dechlorination rate (Fig. 6A and B and Table S3). NDH and SDH catalyze quinone reduction. Rotenone blocks electron transfer between the iron–sulfur clusters in NDH and quinone [64]. However, rotenone treatment resulted in imperceptible differences in both EEU and dechlorination rate (Fig. 6A and B and Table S3). On exposure to thenoyltrifluoroacetone, which inhibits the iron–sulfur cluster in the SdhB subunit of SDH [65], the dechlorination rate and EEU significantly decreased to 25.6 ± 2.2% and 53.6 ± 7.3% of the control values, respectively (*P* < .001; Fig. 6A and B and Table S3). These results suggest that SDH-catalyzed quinone reduction may serve as an important electron hub in coupling EEU with the organohalide-respiratory ETC.

Hydroquinone must be oxidized to transfer electrons. Cytochrome *bc*_1_ can oxidize hydroquinone [66]. Treating cells with antimycin A, which blocks the Q_i_ site of the cytochrome *bc*_1_ complex [67], completely inhibited EEU and TCP reduction (*P* < .001) (Fig. 6A and B and Table S3). In addition, carbonyl cyanide *m*-chlorophenyl hydrazine (CCCP) and dicyclohexylcarbodiimide (DCCD) treatments, which respectively dissipate the proton motive force and inhibit ATP synthase [61], also inhibited EEU and TCP reduction (Fig. 6A and B and Table S3). Taken together with our proteomic data, these results are consistent with a role of cytochrome *bc*_1_ and ATP synthase in reductive dechlorination. The reduction potential required for TCP reduction is much lower than that of cytochrome *bc*_1_ [68, 69]; thermodynamically, it is not feasible to couple the oxidation of cytochrome *bc*_1_ with the reduction of TCP. Reduced ferredoxin is believed to directly power the TCP reduction [29]. Compared with cells without an extracellular electron source, both the electron bifurcation complex Etf(Fix)AB (which catalyzes ferredoxin reduction) and the dehalogenase CprA were expressed at significantly higher levels when the cells performed EEU (Fig. 6C). This result is consistent with the possibility that electron bifurcation contributes to the coupling of EEU and TCP reduction, and suggests a potential electron bifurcation process that couples cytochrome *bc*_1_ oxidation with the generation of reduced ferredoxin. However, none of the chemical inhibitors completely inhibited organohalide-respiratory ETC. This might be due to off-target effects or limitations in their inhibitory activity [70, 71].

**Figure 7:**
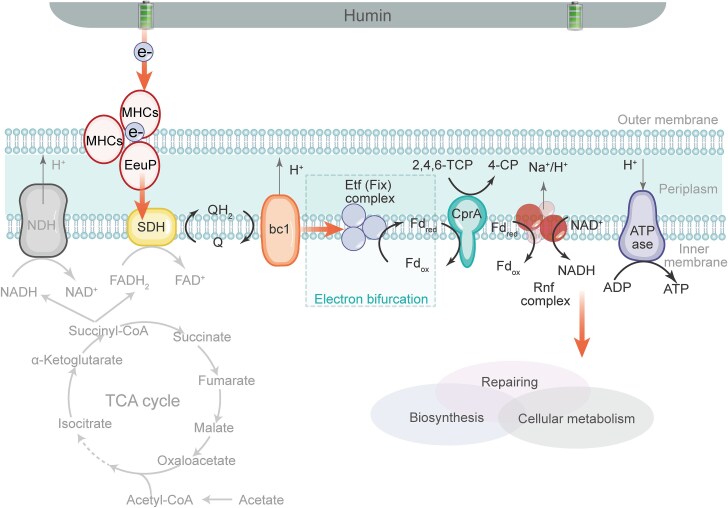
A schematic diagram of the coupling of humin oxidation with organohalide respiration in *Pseudomonas* sp. CP-1. The orange arrows represent electron flow. EeuP oxidizes humin and transfers electrons to inner-membrane succinate dehydrogenase (SDH), which subsequently catalyzes the reduction of quinone (Q). The generated hydroquinone (QH_2_) is oxidized by the cytochrome *bc*_1_ complex (bc_1_), which further transfers electrons to the electron bifurcation complex [Etf (Fix) complex] while establishing a transmembrane proton gradient. The Etf (Fix) complex catalyzes the reduction of ferredoxin (Fd), which in turn powers the reduction of TCP catalyzed by the reductive dehalogenase CprA. Reduced ferredoxin (Fd_red_) can also be oxidized by the Na^+^-translocating ferredoxin:NAD^+^ reductase (Rnf) complex, leading to the generation of a transmembrane sodium gradient. The resulting chemiosmotic gradients drive ATP synthesis. MHCs: multiheme cytochromes; NDH: NADH dehydrogenase; ATPase: ATP synthase.

We illustrate a model of humin oxidation-driven organohalide reductive respiration in *Pseudomonas* sp. CP-1 (Fig. 7): Strain CP-1 directly accepts electrons from humin and transfers electrons with the help of cytochrome EeuP and other cytochromes; the electrons are transported to SDH, which catalyzes the reduction of quinone; hydroquinone is oxidized by cytochrome *bc*_1_ while simultaneously establishing a transmembrane proton gradient; the electrons are then transferred to the electron bifurcation Etf(Fix) complex, which catalyzes the reduction of ferredoxin; the reduced ferredoxin powers the reduction of TCP by reductive dehalogenase CprA, or is oxidized by the Na^+^-translocating ferredoxin:NAD^+^ reductase (Rnf) complex to generate NADH and a transmembrane sodium gradient [29]; the transmembrane osmotic gradients drive the generation of ATP.

## Discussion

Our study reveals an energy acquisition strategy involving humin oxidation in *Pseudomonas* sp. CP-1. Reductive dehalogenation is predominantly an anaerobic process, often occurring in environments with low oxygen levels, which frequently overlap with oligotrophic conditions. The majority of OHRB are heterotrophs that rely on H_2_ or volatile fatty acids [18]. Therefore, to survive in oligotrophic environments, OHRB have mainly adapted to grow syntrophically with other fermentative microorganisms [21]. In this study, we first demonstrated that humin oxidation can support reductive dichlorination in various groundwater ecosystems. We further studied an organohalide-respiring bacterium, *Pseudomonas* sp. CP-1, isolated from TCP-contaminated groundwater ecosystems. Our study demonstrates that strain CP-1 can perform respiratory dehalogenation in different oligotrophic aquifer materials via the direct acceptance of electrons from humin, suggesting that humin serves as an additional electron source accessible to OHRB. Considering the ubiquity of humin in oligotrophic dehalogenation environments such as deep subsurface and groundwater [72], humin is likely a general electron source for the organohalide respiration of OHRB, broadening our knowledge of microbial oligotrophy and organohalide bioremediation.

This study provides the concrete evidence showing that solid-phase humin acts as a direct electron donor to support reductive dehalogenation by OHRB. Generally, humic substances have been considered electron donors that support microbial reduction of various electron acceptors. In particular, humic acid has been widely shown to directly provide electrons to drive various redox processes, such as nitrate reduction, sulfate reduction, Fe(III) reduction, and methanogenesis [24, 51, 52]. In some cases, the carbon skeleton of humic acid is metabolized to serve as an electron donor [73]. However, our data show that humic acid was not used as an electron donor to support reductive dehalogenation by strain CP-1. Humin constitutes the majority of humic substances. It has previously been suggested to function as an electron shuttle or donor, facilitating specific microbial consortia in performing reductive dehalogenation, dissimilatory iron reduction, nitrate reduction, and reductive acetogenesis [27, 28, 51]. In this study, we demonstrate that humin oxidation is linked to the energy metabolism of OHRB, and provide mechanistic details on the coupling of humin oxidation with reductive dehalogenation in the OHRB strain CP-1. Specifically, hydrogen was neither generated nor used during the process (Fig. S2); the EEU pathway was connected to the organohalide-respiratory ETC; and the redox coupling directly contributed to energy metabolism. These results deepen our understanding of the ecological functions of humin. However, humin primarily serves as an electron donor, rather than as carbon source for OHRB. Therefore, its oxidation should only be an adaptive strategy for OHRB to survive in oligotrophic environments while supplying labile carbon readily promotes heterotrophic OHRB activity in those bioremediation sites [21, 22].

This study identifies a EEU pathway consisting of transmembrane cytochrome EeuP and SDH. Porin–cytochrome complexes (e.g. Mtr, Omc-Pcc, and Pio), which are composed of porin proteins and multiple cytochromes, coupled with inner membrane cytochromes, have been recognized as key structures that enable microorganisms to take up electrons from extracellular electron donors [61, 74–76]. Porin–cytochrome protein complexes are usually encoded by gene clusters in the genome. However, genomic analyses indicated that strain CP-1 lacks homologous genes for porin–cytochrome protein complexes. By contrast, our results showed that CP-1 uses a transmembrane cytochrome, EeuP, to take up electrons. In particular, EeuP forms a distinct branch separate from porin–cytochrome protein complexes in a phylogenetic tree (Fig. S13). However, EeuP only contains four hemes, which may not be sufficient to mediate electron transfer across the cell membrane [77]. The structure of EeuP predicted by AlphaFold3 showed an N-terminal transmembrane helix and a C-terminal extracellular domain containing all four hemes (Fig. S14). Compared with the other outer membrane electron uptake cytochrome, it exhibits a compact architecture and has fewer predicted surface-exposed regions potentially involved in protein–protein interaction. We speculate that EeuP could directly oxidize humin but needs to cooperate with other periplasmic cytochromes to facilitate electron uptake. Furthermore, our results demonstrate that deletion of EeuP significantly, though not completely, inhibits EEU or TCP dechlorination. Given that the *Pseudomonas* sp. CP-1 genome encodes additional transmembrane cytochromes (Data S5), which were also upregulated in humin-oxidation cells, we cannot rule out the possibility that they may also contribute, at least in part, to EEU. These findings warrant further study. Previous studies have indicated that the ubiquinol: cytochrome *c* oxidoreductase complex is the primary site for the conversion of electrical energy into chemical energy (reduced quinone) [67]. In contrast, our data show that SDH catalyzes this conversion in *Pseudomonas* sp. CP-1, at least when the terminal electron acceptor is an organohalide, providing new perspectives on microbial electrotrophy.

Even though *Pseudomonas* species are not typical OHRB, the conservation of EeuP homologs in *Pseudomonas* should contribute to their broad metabolic versatility (Fig. 5A and S11A). *Pseudomonas* are frequent occurrence in oligotrophic environments. The expression of EeuP may modulate electron flow and support anaerobic respiration, such as using quinones or nitrate as terminal electron acceptors, and finally contributes to the ecological fitness of *Pseudomonas* in redox-stratified and oligotrophic environments. The phylogenetic proximity of clade II to clade I implies either an early shared ancestor or ancient horizontal gene transfer events. Furthermore, given that most strains within clade II also inhabit redox-gradient environments (e.g. aquatic systems), EeuP distribution may reflect convergent functional adaptation to these niches. In contrast, the distant phylogenetic positioning of clade III (OHRB) relative to clade I indicates the later acquisition of *eeuP* by OHRB via horizontal transfer, likely conferring fitness advantages in their native oligotrophic habitats. Significantly, clade III exhibits complex branching patterns and long branch lengths, with an average distance of 2.13 substitutions/site—markedly higher than the other clades. This indicates that *eeuP* likely underwent multiple independent horizontal gene transfer events within OHRB lineages. Phylogenetic examination of EeuP distributions in OHRB also identified highly similar EeuP sequences in ecologically analogous but evolutionarily distant OHRB (e.g. *Sulfurospirillum* and *Dehalogenimonas*), further supporting the likelihood of horizontal gene transfer among functionally convergent microorganisms. Significantly, EeuP homologs are also widely distributed among bacteria capable of respiring oxidized sulfur species, as well as iron-reducing and nitrate-reducing bacteria (Fig. S11), which are commonly found in oligotrophic environments. In addition, metagenomic analysis of microbiomes from other typical oligotrophic environments, such as groundwater, marine sediments, and peatlands, also revealed the widespread distribution of EeuP homologs (Fig. S15 and Table S4). These results suggest that EEU might be an adaptive mechanism used by EeuP-equipped non-OHRB to survive in oligotrophic environments. It warrants further verification.

Proteomic data showed that proteins related to flavoprotein synthesis and flavin secretion were significantly upregulated in strain CP-1 when the cell respired with humin. As expected, significantly higher levels of flavin were detected both inside and outside cells oxidizing humin compared with those metabolizing acetate (Fig. S16). Previous studies indicated the possibility that flavin mediates the electron transfer between extracellular electron donors and bacterial cells [78]. No significant redox peak assigned to free-form flavin (−0.27 V) could be detected during EEU (Fig. S17). However, two redox peaks with midpoint potentials of −0.30 V and − 0.07 V were detected according to differential pulse voltammetry (DPV) and cyclic voltammetry (CV) results (Fig. S17). Previous studies also indicated that flavin can be bound as a redox cofactor of cytochromes to accelerate extracellular electron transfer and the redox peak of flavin shifts after binding [79]. Our study also showed all cells were attached to humin (Fig. S18). Therefore, we propose that flavin may participate in EEU as a bound redox cofactor.

Supplying electron donors is an efficient strategy to augment microbial reductive dehalogenation. Small organic acids such as acetate and lactate, as well as hydrogen, have been added in the field to directly drive the organohalide respiration of OHRB [24, 80]. Our study identifies humin as an alternative electron source for microbial dehalogenation. Considering its stability, durability, and high electron storage capacity, the addition of humin could serve as a promising strategy for bioremediation of organohalide-contaminated environments.

## Supplementary Material

Supplementary_Data_wraf207

Supplementary_Materials_wraf207

## Data Availability

All data are available in the main text or the supplementary materials. Proteomic data are available via ProteomeXchange with identifier PXD061683.
