# Supplementary material for: Humin oxidation drives microbial dehalogenation in oligotrophic environments
Source: ISME J. 2025 Sep 17;19(1):wraf207. doi: 10.1093/ismejo/wraf207 (PMC12503164; doi:10.1093/ismejo/wraf207)
Supplement: Supplementary_Materials_wraf207 [file supplementary_materials_wraf207.docx]

**Supplementary Materials for**

**Humin oxidation drives microbial dehalogenation in oligotrophic environments**

**Authors**

Zimeng Zhang ^1,#^, Xing Liu ^2,#^, Zhiling Li ^1,*^, Xueqi Chen ^1^, Yunxia Zu ^1^, Shih-Hsin Ho ^1^, Bin Liang ^3^, Shungui Zhou ^2^, Aijie Wang ^1,3,*^

**Affiliations**

^1^ State Key Laboratory of Urban-rural Water Resource and Environment, School of Environment, Harbin Institute of Technology, Harbin, Heilongjiang 150090, China

^2^ Fujian Provincial Key Laboratory of Soil Environmental Health and Regulation, College of Resources and Environment, Fujian Agriculture and Forestry University, Fuzhou, Fujian 350002, China

^3^ School of Eco-Environment, Harbin Institute of Technology (Shenzhen), Shenzhen, Guangdong 518055, China

^#^ These authors contributed equally: Zimeng Zhang, Xing Liu.

*Corresponding author. Email: lizhiling@hit.edu.cn (Z.L.); waj0578@hit.edu.cn (A.W.)

**This PDF file includes:**

Supplementary Methods Text S1 to S5

Figs. S1 to S18

Tables S1 to S4

### **Text S1.** Extraction and characterization of humic substances.

Humic substances were isolated from 100 g of aquifer materials (sieved to < 2 mm) under strictly anoxic conditions (H_2_O/O_2_ < 0.1 ppm) in an anaerobic glove box (YQX-II, Shanghai Yuejin, China) using ultrapure nitrogen, following modified procedures by International Humic Substances Society (IHSS) [1, 2]. All distilled water and solutions were purged with ultrapure nitrogen for 30 minutes prior to use. Samples were agitated at 150 rpm in 150 mL of 2.5 M hydrofluoric acid (HF) solution for 16 h to remove silicate and clay minerals [3], followed by centrifugation (8000 g, 10 min, 4 °C). After decanting the supernatant, the procedure was repeated with progressively increased shaking durations: three cycles of 24 h, two cycles of 48 h, and one cycle of 64 h, to ensure complete mineral removal. The collected supernatant was used for fulvic acid extraction by an XAD-8 resin adsorption method [1], followed by rotary evaporation (40 °C, 150 mbar) and lyophilization [4]. The residues underwent alkaline extraction with 50 mL of 6 M NaOH for 12  h and were then repeatedly washed with 0.1 M NaOH (at least10 times) until the supernatant showed negligible absorbance at 380 nm [5]. The precipitate (humin) was treated with three successive cycles of 24-hour agitation in 200 mL of 1 M HF, followed by rinsing with water. Acidification of the alkaline supernatant to pH < 2 using 2 M HCl (at 150 rpm for 12 h) yielded humic acid (HA), which was recovered by centrifugation and washed at least three times to remove residual chloride. All finial products were lyophilized for 24 h using a 2KBTXL freeze dryer (VirTis, USA) and quantified gravimetrically based on dry weight.

### **Text S2.** Preparation of MSM medium.

The mineral salts medium (MSM) comprised 0.2 g L^-1^ KH_2_PO_4_, 0.4 g L^-1^ Na_2_HPO_4_ 12H_2_O, 0.3 g L^-1^ NaHCO_3_, 0.001 g L^-1^ CaCl_2_·2H_2_O, 0.05 g L^-1^ MgCl_2_, 0.13 g L^-1^ NH_4_Cl, 0.1 mL L^-1^ SL-10 trace element solution, and 1.0 mL L^-1^ vitamin concentrate. The SL-10 trace element solution contained 30 g L^-1^ MgSO_4_·7H_2_O, 5 g L^-1^ MnSO_4_·H_2_O, 1 g L^-1^ FeSO_4_·7H_2_O, 1 g L^-1^ CoCl_2_·6H_2_O, 1 g L^-1^ ZnSO_4_·7H_2_O, 0.1 g L^-1^ CuSO_4_·5H_2_O, 0.1 g L^-1^ AlK(SO_4_)_2_·12H_2_O, 0.1 g L^-1^ H_3_BO_3_ and 0.1 g L^-1^ Na_2_MoO_4_·2H_2_O. The vitamin concentrate included 20 mg L^-1^ biotin, 20 mg L^-1^ folic acid, 100 mg L^-1^ pyridoxine hydrochloride, 50 mg L^-1^ thiamine, 50 mg L^-1^ riboflavin, 50 mg L^-1^ niacin, 50 mg L^-1^ calcium pantothenate, 1 mg L^-1^ Vitamin B12, 50 mg L^-1^ *p*-aminobenzoic acid and 50 mg L^-1^ lipoic acid [6]. All chemicals were obtained from Aladdin Industrial Corporation (Shanghai, China) and were of analytical grade. Aqueous solutions were prepared using ultrapure deionized water (Milli-Q system, 18.2 MΩ·cm at 25 ± 2 °C; Molelement, Chongqing Moore Water Treatment Equipment Co., China).

### **Text S3.** Quantitative reverse transcription PCR analysis.

The expression of genes encoding the electron transport complex involved in electron bifurcation (Etf complex) and reductive dehalogenase (CprA) were quantified using quantitative reverse transcription PCR (qRT-PCR, CFX-96, Bio-Rad, Singapore). This analysis adhered to the MIQE (Minimum Information for Publication of Quantitative Real-Time PCR Experiments) guidelines and EMMI (Environmental Microbiology Minimum Information) recommendations [7, 8].

RNA from aquifer materials was extracted using a commercial soil RNA extraction kit (Sangon Biotech, China) following the manufacturer’s protocol. RNA from strain CP-1 was extracted using the UNlQ-10 Column Trizol Total RNA Isolation Kit (B511321, Sangon Biotech, China), also following the manufacturer’s instructions. Residual DNA was removed using gDNA digester Mix (Sangon Biotech, China) according to the manufacturer’s protocol. RNA concentration and purity were measured using a NanoDrop 2000 spectrophotometer (Thermo Scientific, USA), and integrity was evaluated with a Bioanalyzer. An exogenous RNA spike-in control (ERCC RNA Spike-In Mix, Thermo Fisher Scientific) was added at 1×10^5^ copies per sample during lysis to monitor RNA extraction efficiency, with acceptable recovery rates defined as 70–120%. Reverse transcription to cDNA was performed using the MightyScript Plus First Strand cDNA Synthesis Master Mix (B639252, Sangon Biotech, China) following the manufacturer’s instructions. Briefly, the 20-μL reaction contained 1 μg of DNase-treated RNA, dissolved in 15 μL RNase-free water, along with 5 μL of M-MLV Reverse Transcriptase. The thermal program included 5 min at 25 °C, 15 min at 60 °C, and 5 min at 85 °C for enzyme inactivation, followed by immediate cooling on ice. The resulting cDNA stored at −80 °C until further use.

Quantitative PCR (qPCR) was performed using SYBR Green I dye with the SGExcel FastSYBR Mixture kit (Sangon Biotech, China) on a CFX96 system. The thermal cycling protocol consisted of an initial denaturation at 95 °C for 3 min, followed by 40 cycles of 95 °C for 5 s and 60 °C for 20 s. Melt curve analysis was conducted at 95 °C for 15 s, followed by 60 °C for 60 s, with a gradual increase to 95 °C at 0.5 °C increments. The 20-μL reaction mixture contained 10 μL of 2× SYBR mixture, 0.4 μL of each primer, 1 μL of cDNA template, and 8.2 μL of RNase-free water. No-template controls (NTCs) which substituted cDNA with nuclease-free water, were included in each run to monitor for contamination. No-reverse transcription (no-RT) controls, prepared by omitting reverse transcriptase during cDNA synthesis, were used to assess DNA contamination. Data were only accepted when NTCs and no-RT controls showed undetermined results.

For absolute quantification of *cprA* gene in environmental samples, DNA from the aquifer material was extracted first using the FastDNA Spin Kit for Soil (MP Biomedicals, CA, USA) according to the manufacturer’s protocol. This DNA served as the template for amplifying the *cprA* gene using the primer pair Dehalo-F/Dehalo-R. The resulting DNA fragment was cloned into the pUCI-Blunt Zero cloning vector (Sangon Biotech, China) via blunt-end cloning, following the manufacturer’s instructions. The resulting plasmid, pUCI-Dehalo, was verified by sequencing. The standard curve for *cprA* was constructed using ten-fold serial dilutions of pUCI-Dehalo, ranging from 10^4^ to 10^9^ copies/μL.

Genomic DNA of strain CP-1 was separated using TIANcombi DNA Lyse&Det PCR Kit (TIANGEN Biotech, Beijing, China). For absolute quantification of *fixA*, *fixB* and *cprA* expression in stain CP-1, the corresponding genes were amplified from the strain CP-1 genome using primer pairs of *fixA*-F/*fixA*-R, *fixB*-F/*fixB*-R and *cprA*-F/*cprA*-R, respectively. The amplified fragments were individually cloned into the pUC-Blunt Zero cloning vector, yielding the plasmids pUCI-*fixA*, pUCI-*fixB* and pUCI-*cprA*, which served as quantification standards.

### **Text S4.** ∆*eeuP* mutant strain construction.

The *eeuP* knockout mutant (Δ*eeuP*) was constructed by homologous recombination using a suicide vector strategy [9]. Approximately 1 kb fragments upstream and downstream of *eeuP* were PCR-amplified from *Pseudomonas* sp. CP-1 genomic DNA with high‑fidelity DNA polymerase, and the gentamicin resistance cassette (*aacC1*) was amplified from plasmid pJQ200SK. These three fragments were assembled via overlap extension PCR to generate the Δ*eeuP*::Gm construct, which was cloned into the suicide vector pCVD442 by restriction-ligation, yielding pCVD442-Δ*eeuP*::Gm. The recombinant plasmid was introduced into *E. coli* β2155 by electroporation and subsequently transferred into *Pseudomonas* sp. CP-1 by biparental conjugation. Transconjugants were selected on gentamicin-containing plates, and single-crossover integrants (CP-1/pCVD442-Δ*eeuP*::Gm) were resolved by sucrose counterselection on LB agar supplemented with 10% sucrose. Double‑crossover mutants (CP‑1Δ*eeuP*::Gm), in which the *eeuP* locus was replaced by the gentamicin resistance cassette, were confirmed by PCR screening.


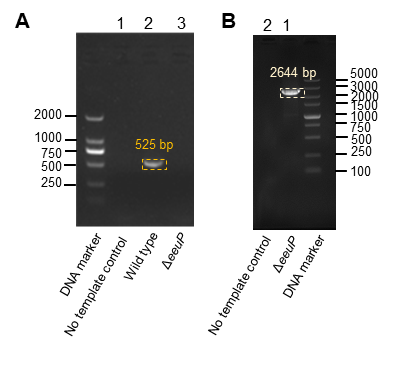


**Δ*eeuP* verification.** Agarose gel electrophoresis of PCR products amplified using (A) primer pair *eeuP*-inF/*eeuP*-inR and (B) primer pair *eeuP*-outF/*eeuP*-outR. All tested strains are labelled under the individual lanes. The numbers on the DNA bands indicate the size of the resulting PCR products.

### **Text S5.** Pseudo-first-order kinetics of dechlorination

The reduction of 2,4,6-trichlorophenol (2,4,6-TCP) follows the pseudo-first-order kinetic model, expressed as:

Where [TCP] is the TCP concentration (μM) at time t, and *k* is the pseudo-first-order rate constant (d^-1^). Integration of this equation yields:

Where [TCP]_0_ is the initial TCP concentration. The pseudo-first-order rate constant *k,* which reflects the TCP reduction rate [10–12], was determined through nonlinear regression using a one-phase exponential decay model in GraphPad Prism 9.3 (GraphPad Software, USA).

***Reference***

1. Thurman EM, Malcolm RL. Preparative isolation of aquatic humic substances. *Environ Sci Technol* 1981; **15**:463–466. https://doi.org/10.1021/es00086a012

2. Huang F, Liu H, Wen J, et al. Underestimated humic acids release and influence on anaerobic digestion during sludge thermal hydrolysis. *Water Res* 2021; **201**:117310. https://doi.org/10.1016/j.watres.2021.117310

3. Zhang C, Katayama A. Humin as an electron mediator for microbial reductive dehalogenation. *Environ Sci Technol* 2012; **46**:6575–6583. https://doi.org/10.1021/es3002025

4. Xue S, Xiao Y, Wan K, et al. The fractionation of fulvic acid and the optimal fraction as explanatory factors for binding characteristics of lead in aqueous solution. *Sep Purif Technol* 2021; **275**:119061. https://doi.org/10.1016/j.seppur.2021.119061

5. Yan M, Mo S, Liu Z, et al. Absorptivity inversely proportional to spectral slope in CDOM. *Environ Sci Technol* 2025; **59**:7156–7164. https://doi.org/10.1021/acs.est.5c01019

6. Lin X-Q, Li Z-L, Liang B, et al. Accelerated microbial reductive dechlorination of 2,4,6-trichlorophenol by weak electrical stimulation. *Water Res* 2019; **162**:236–245. https://doi.org/10.1016/j.watres.2019.06.068

7. Bustin SA, Benes V, Garson JA, et al. The MIQE guidelines: minimum information for publication of quantitative real-time PCR Experiments. *Clin Chem* 2009; **55**:611–622. https://doi.org/10.1373/clinchem.2008.112797

8. Borchardt MA, Boehm AB, Salit M, et al. The Environmental Microbiology Minimum Information (EMMI) guidelines: qPCR and dPCR quality and reporting for environmental microbiology. *Environ Sci Technol* 2021; **55**:10210–10223. https://doi.org/10.1021/acs.est.1c01767

9. Zheng W, Xia Y, Wang X, et al. Precise genome engineering in *Pseudomonas* using phage-encoded homologous recombination and the Cascade–Cas3 system. *Nat Protoc* 2023; **18**:2642–2670. https://doi.org/10.1038/s41596-023-00856-1

10. Pavlostathis SG, Prytula MT. Kinetics of the sequential microbial reductive dechlorination of hexachlorobenzene. *Environ Sci Technol* 2000; **34**:4001–4009. https://doi.org/10.1021/es991214n

11. Okutman Tas D, Thomson IN, Löffler FE, et al. Kinetics of the microbial reductive dechlorination of pentachloroaniline. *Environ Sci Technol* 2006; **40**:4467–4472. https://doi.org/10.1021/es052103t

12. Shi C, Tong M, Cai Q, et al. Electrokinetic-enhanced bioremediation of trichloroethylene-contaminated low-permeability soils: mechanistic insight from spatio-temporal variations of indigenous microbial community and biodehalogenation activity. *Environ Sci Technol* 2023; **57**:5046–5055. https://doi.org/10.1021/acs.est.3c00278


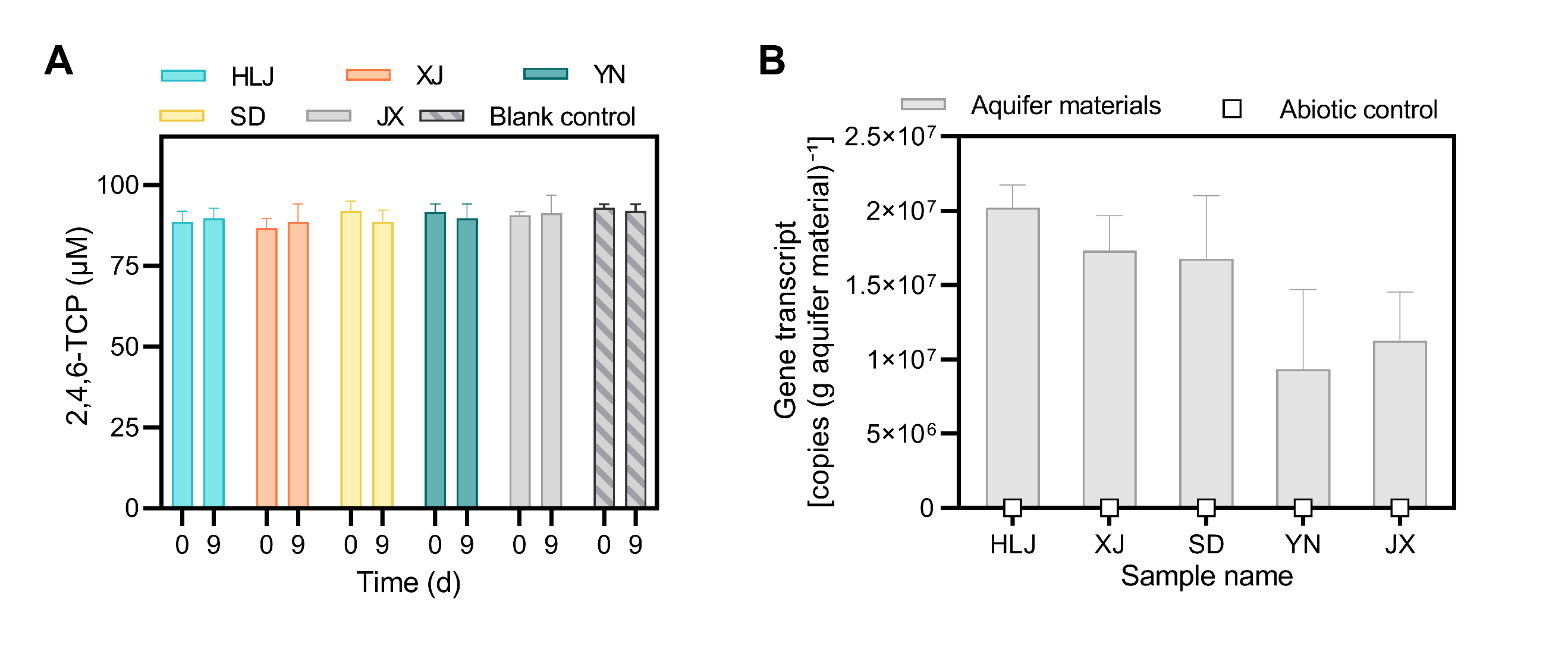


### **Figure S1. Biological reductive dichlorination in five aquifer materials.** **(A)** The reduction of 2,4,6-trichlorophenol (2,4,6-TCP). Under abiotic conditions, TCP remained intact in microcosms. Samples were collected from five provinces in Chinese: Heilongjiang (HLJ, 44°23′N, 127°41′E), Xinjiang (XJ, 36°25′N, 117°34′E), Shandong (SD, 36°25′N, 117°34′E), Yunnan (YN, 24°16′N, 98°11′E), and Jiangxi (JX, 28°34′N, 117°29′E). **(B)** The transcript levels of dehalogenase gene *cprA* in different TCP reduction microcosms after 9 days incubation. Gene transcripts were quantified using absolute quantification, calculated against a standard made from cloned *cprA* amplified from aquifer material. MSM medium containing 100 μM TCP served as a blank control. The abiotic control was prepared by autoclaving the inoculated culture. Data are means ± standard deviation from three biological replicates.

**
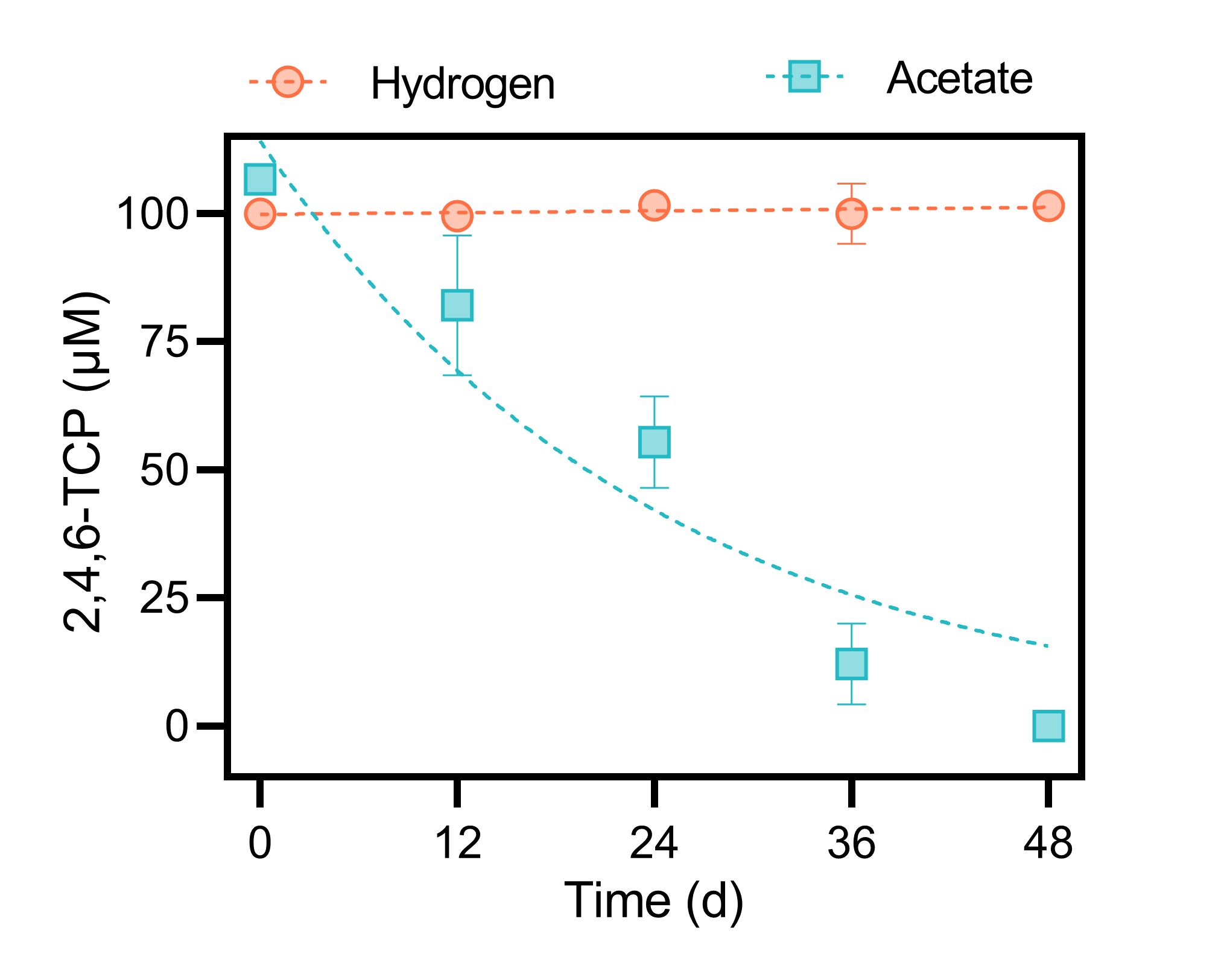
**

### **Figure S2.** **Reduction of 2,4,6-trichlorophenol by strain CP-1 using either acetate (5 mM) or hydrogen (0.5 mmol) as the electron donor.** Data are presented as means ± standard deviation from three biological replicates.


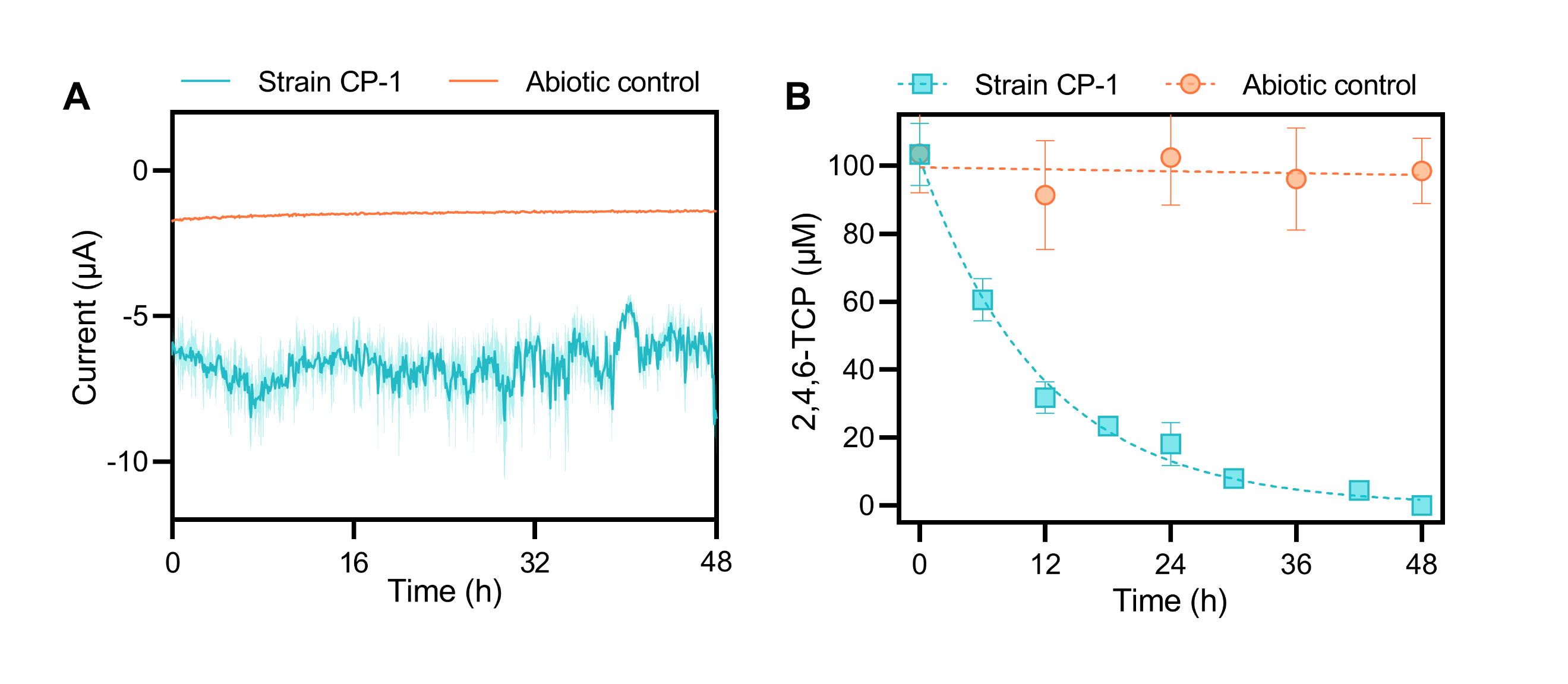


### **Figure S3. Synergistic extracellular electron uptake and TCP reduction in strain CP-1. (A)** The extracellular electron uptake. **(B)** The reduction of 2,4,6-trichlorophenol. The shaded area indicates the standard deviation from three biological replicates. Data are presented as means ± standard deviation from three biological replicates.


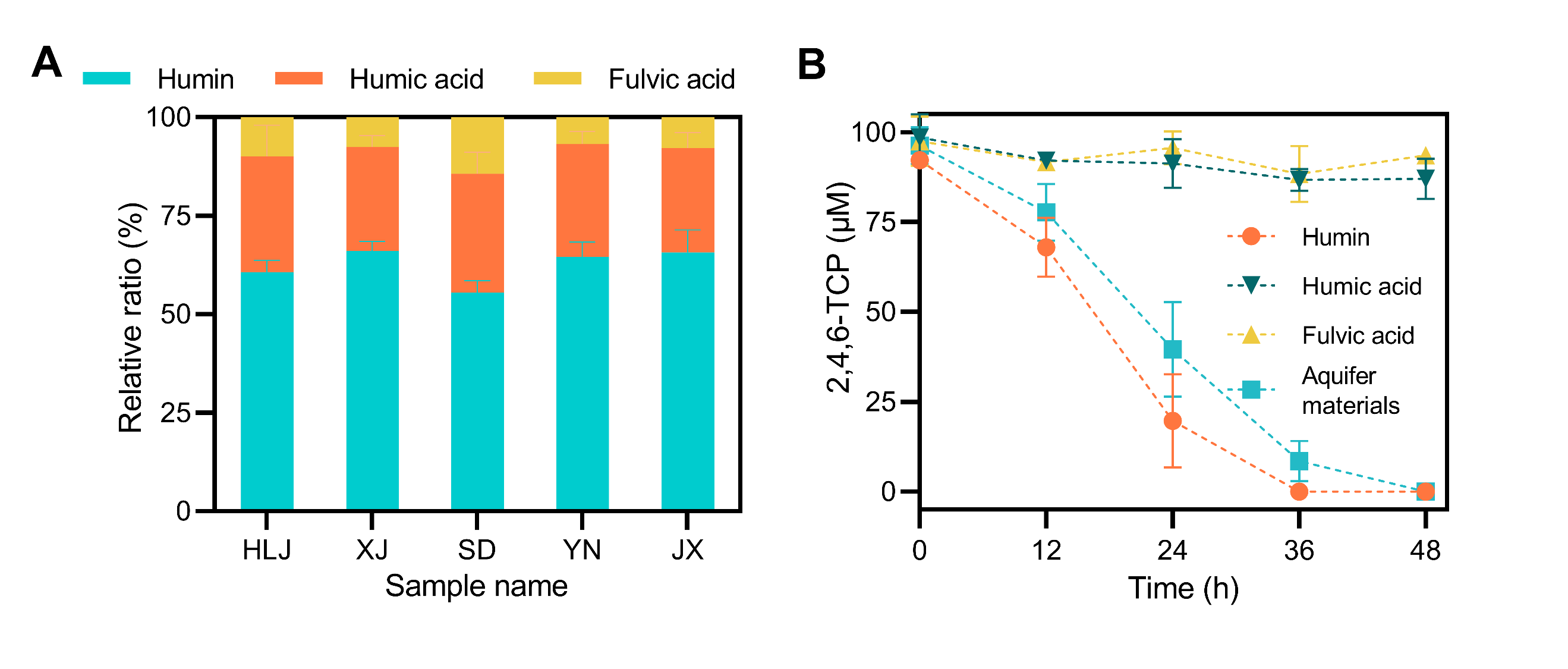


### **Figure S4.** **Strain CP-1 utilizes humin but not humic acid or fulvic acid for reductive dechlorination.** (**A**) Relative proportions of humin, humic acid, and fulvic acid in humic substances, based on dry weight. (**B**) Effects of different humic substances components (5 g L^-1^) on reductive dechlorination by strain CP-1 (~9.4 ± 2.3 ×10^6^ CFU mL^-1^). Data are represented as means ± standard deviation from three biological replicates.


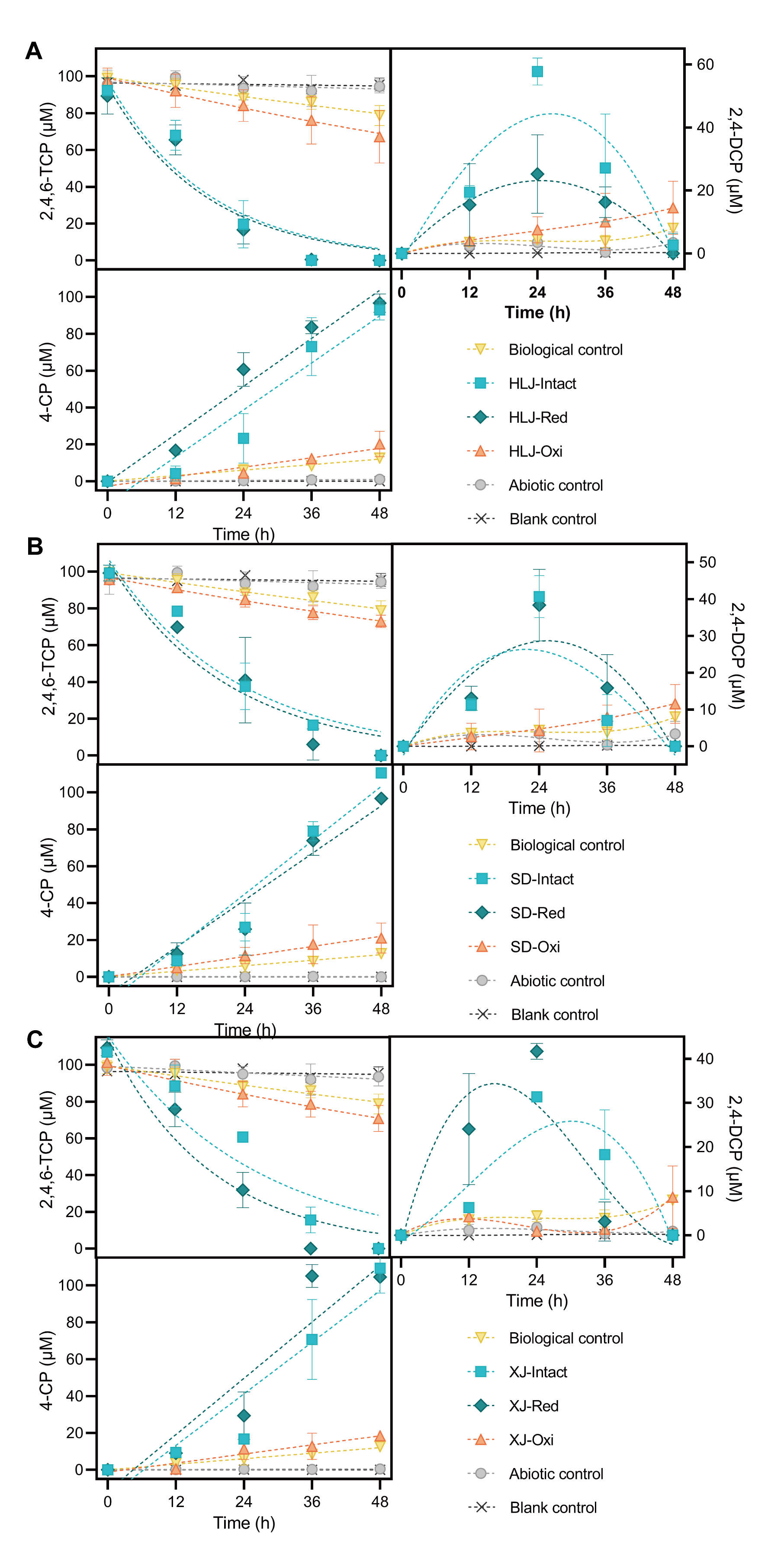

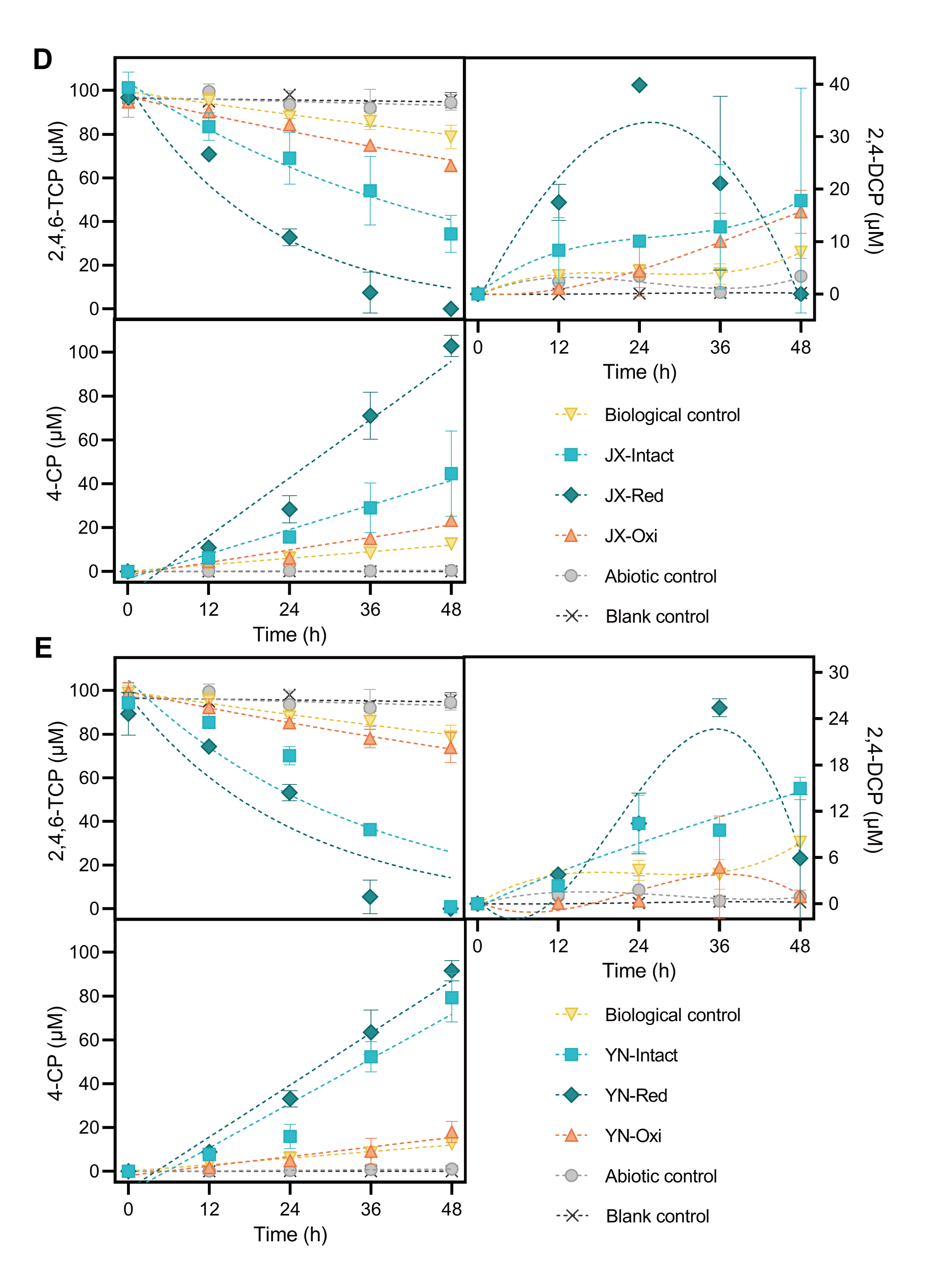


### **Figure S5. Reductive dechlorination kinetics of 2,4,6-TCP mediated by humin extracted from different geographic regions.** Humins (-intact) isolated from aquifers in five Chinese provinces: Heilongjiang (HLJ), Xinjiang (XJ), Shandong (SD), Jiangxi (JX), and Yunnan (YN). Humin was oxidized by 30% (m/v) H_2_O_2_ (6 h, 25 ± 2 °C) or reduced by 0.1 M NaBH_4_ (15 h) under nitrogen-purged anaerobic conditions, yielding oxidized humin (-Oxi) or reduced humin (-Red), respectively. The abiotic control consists of autoclaved humin without inoculum, while the biological control includes only the cell inoculum without humin. MSM medium containing 100 μM TCP served as a blank control. Data are represented as means ± standard deviation from three biological replicates.


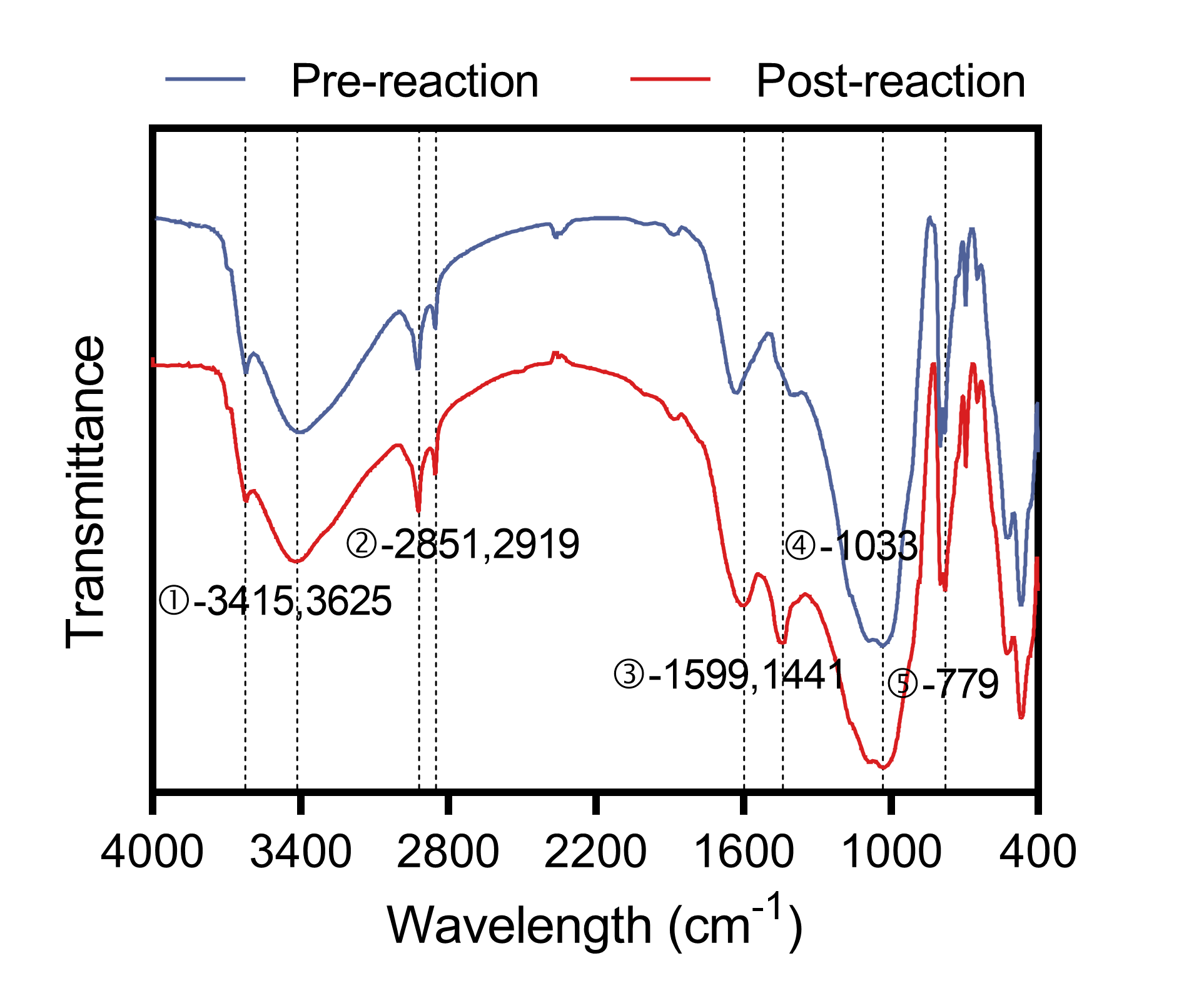


### **Figure S6. Fourier transform infrared spectroscopic characterization of humin.** Humin samples taken before (Pre-reaction) and after (Post-reaction) organohalide respiration of strain CP-1 were analyzed separately. The spectrum displays characteristic absorption bands corresponding to functional groups: (1) 3415 and 3625 cm^−1^ (O-H and N-H stretching vibrations), (2) 2851 and 2919 cm^−1^ (C-H stretching of CH_2_ group), (3) 1599 and 1441 cm^−1^ (C=O and C=N stretching in quinoid structures), (4) 1033 cm^−1^ (C-O stretching and Si-O of silicates), and (5) 779 cm^−1^ (aromatic C–H bending).


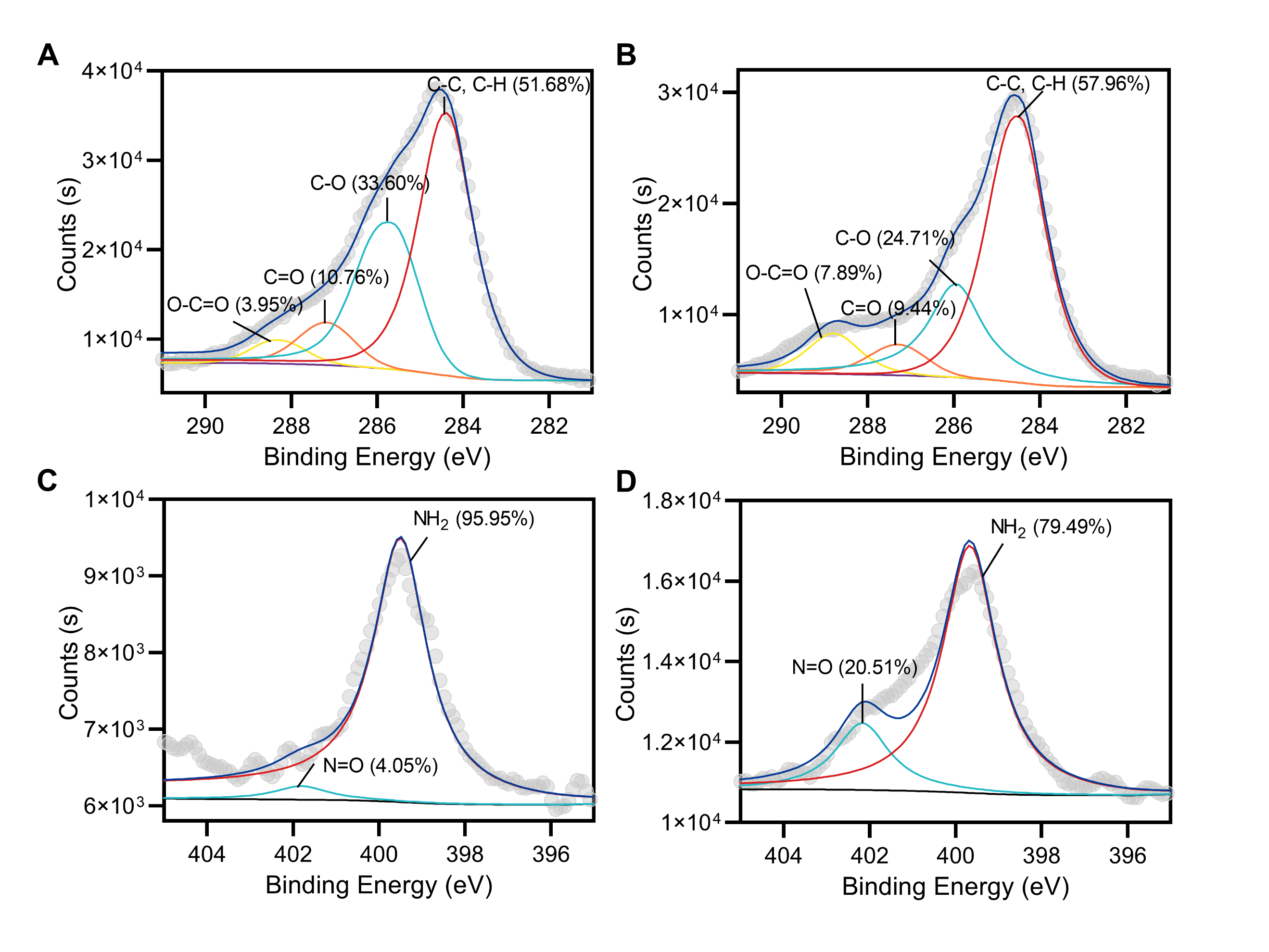


### **Figure S7.** **X-ray photoelectron spectroscopy (XPS) characterization of humin.** C 1s spectra of intact humin **(A)** before and **(B)** after dechlorination; N 1s spectra **(C)** before and **(D)** after dechlorination.


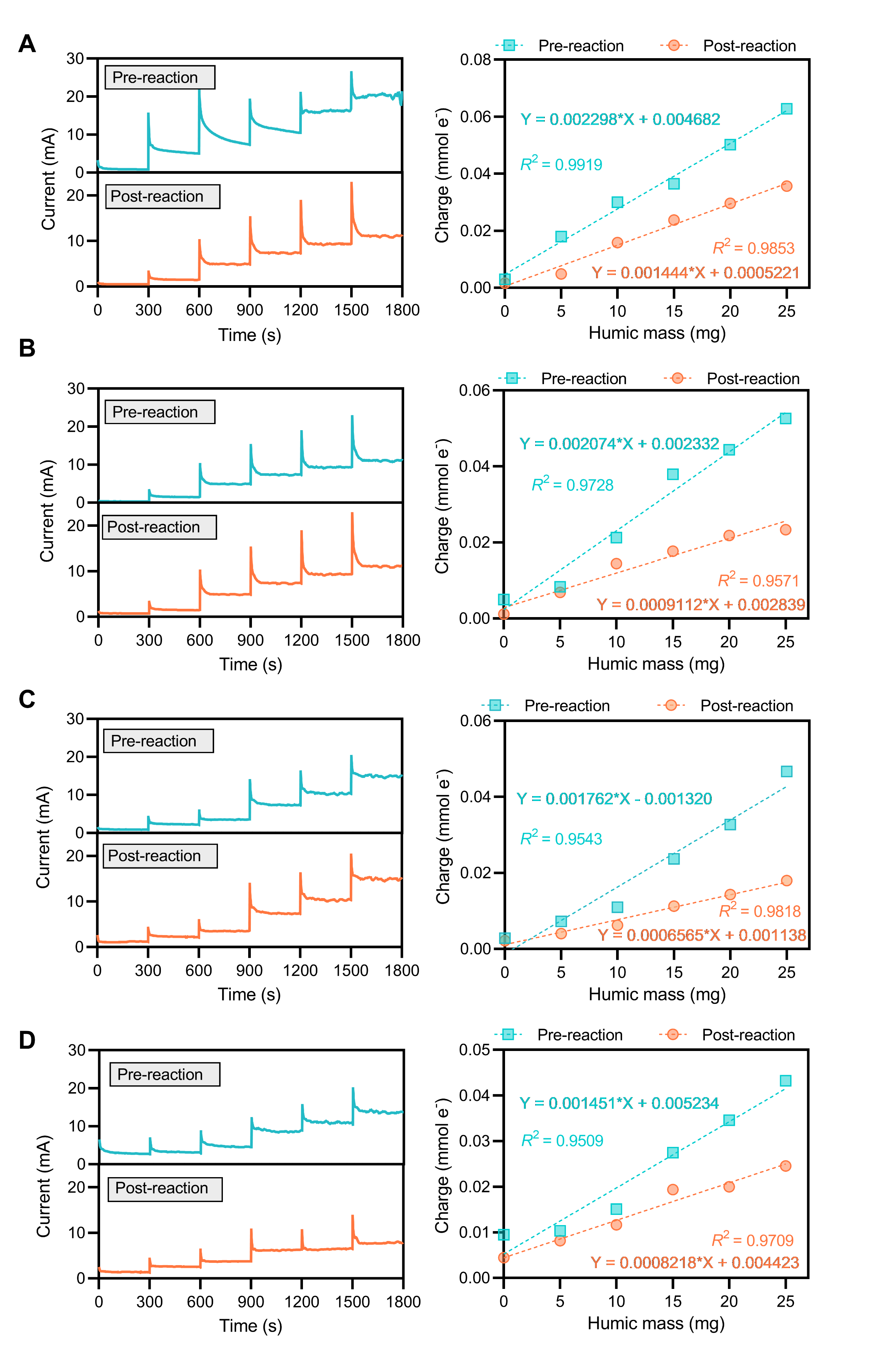

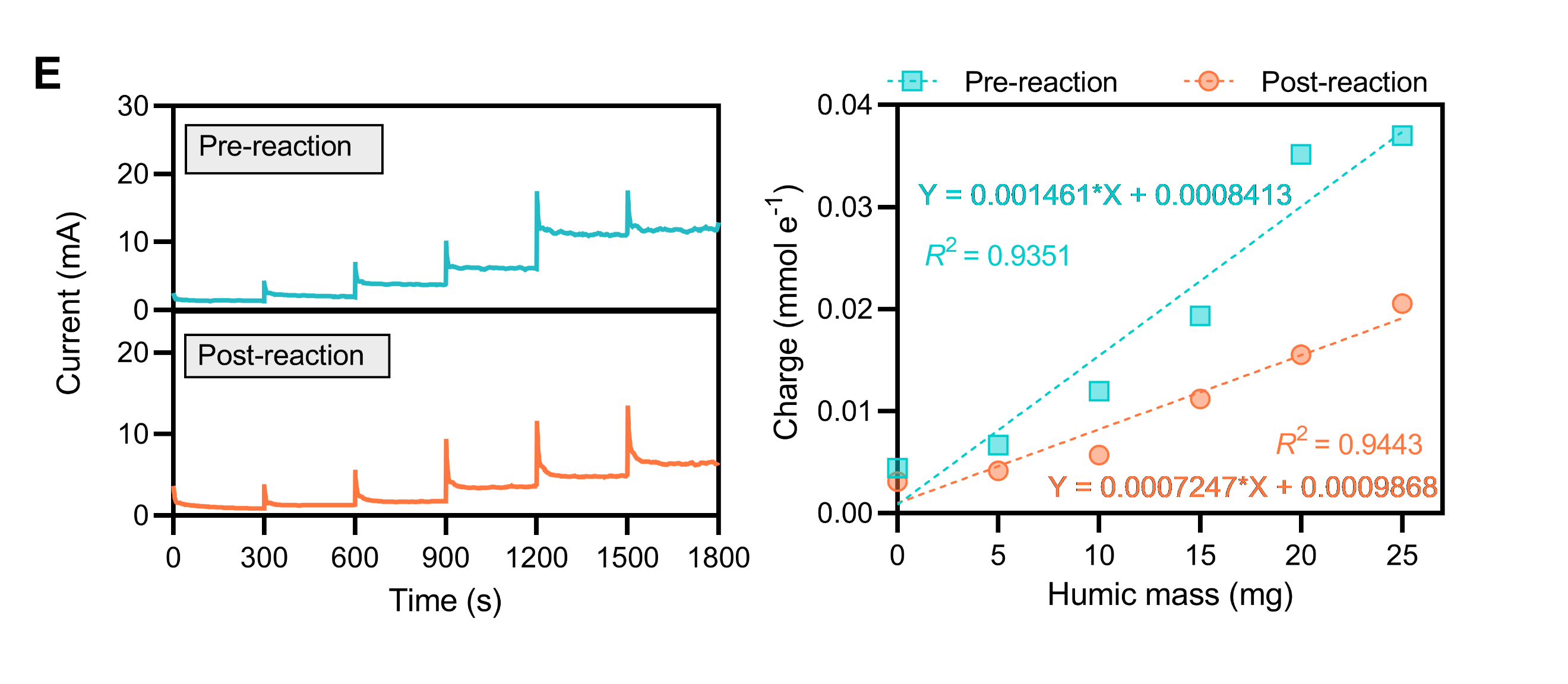


### **Figure S8. Electrochemical characterization of the electron donating capacity (EDC) of humin.** Humin samples from Heilongjiang **(A)**, Shandong **(B)**, Xinjiang **(C),** Jiangxi **(D)**, and Yunnan **(E)** before (Pre-reaction) and after (Post-reaction) organohalide respiration by strain CP-1 were analyzed separately. Left panels: Oxidative current measured at 0.61 V. Right panels: Relationship between humin mass and total released charges. The slope of the linear regression represents the charge released per unit mass of humin, defined as its EDC.


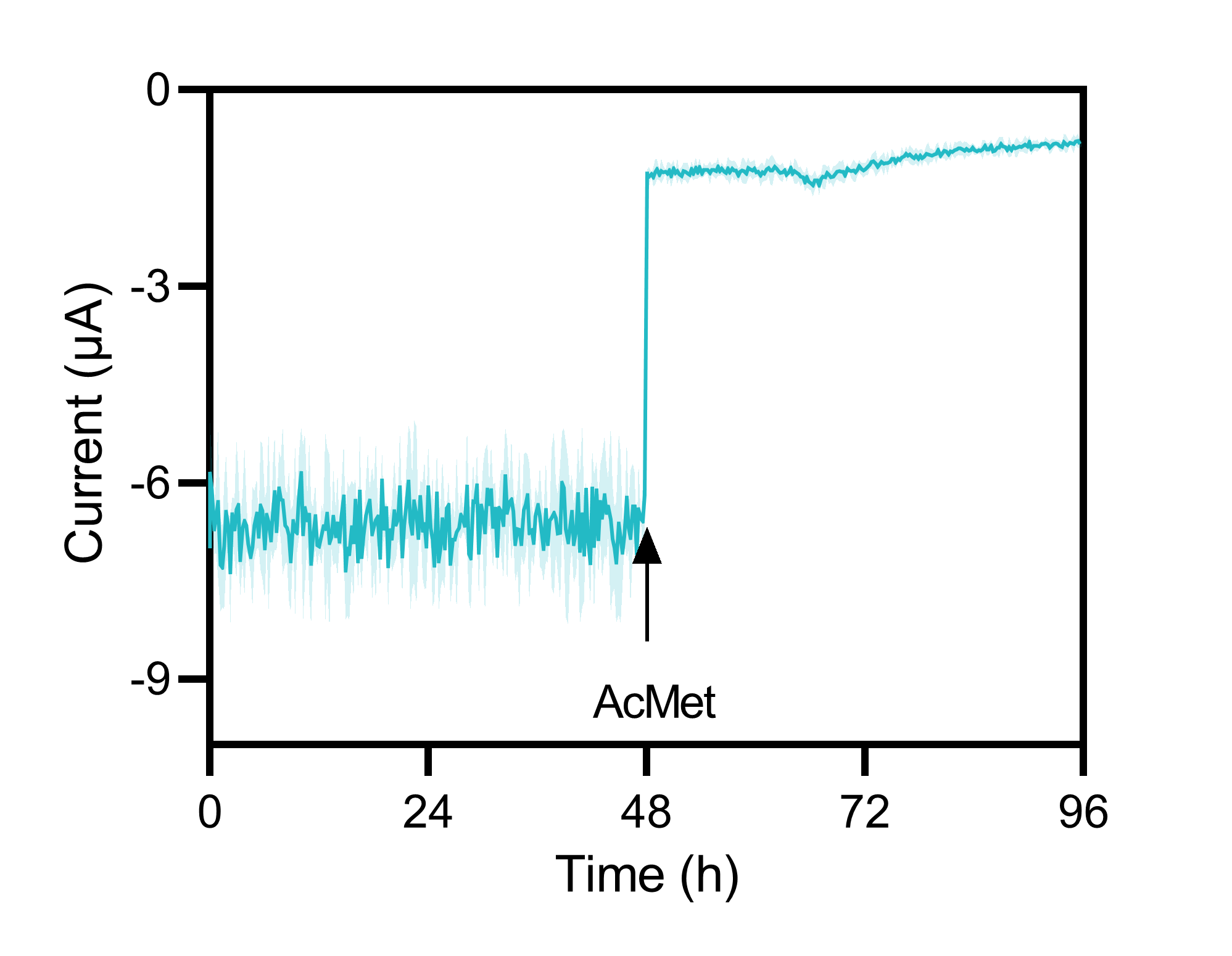


### **Figure S9. Characterization of extracellular electron uptake in strain CP-1.** The cathode was poised at -0.5 V. The shaded area indicates the standard deviation from three biological replicates. The addition of 100 μM acetyl methionine (AcMet) inhibits extracellular electron uptake.


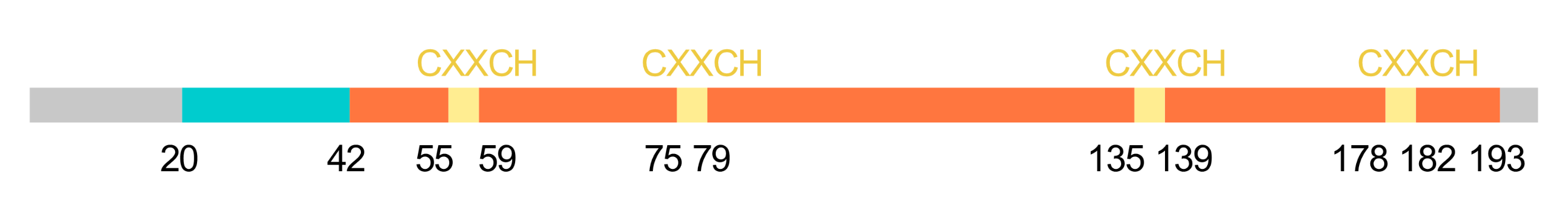


### **Figure S10. Distribution of heme-binding motifs in EeuP.** Putative functional domains as follows: transmembrane helix (cyan), cytochrome c domain (orange), heme-binding motif (yellow).


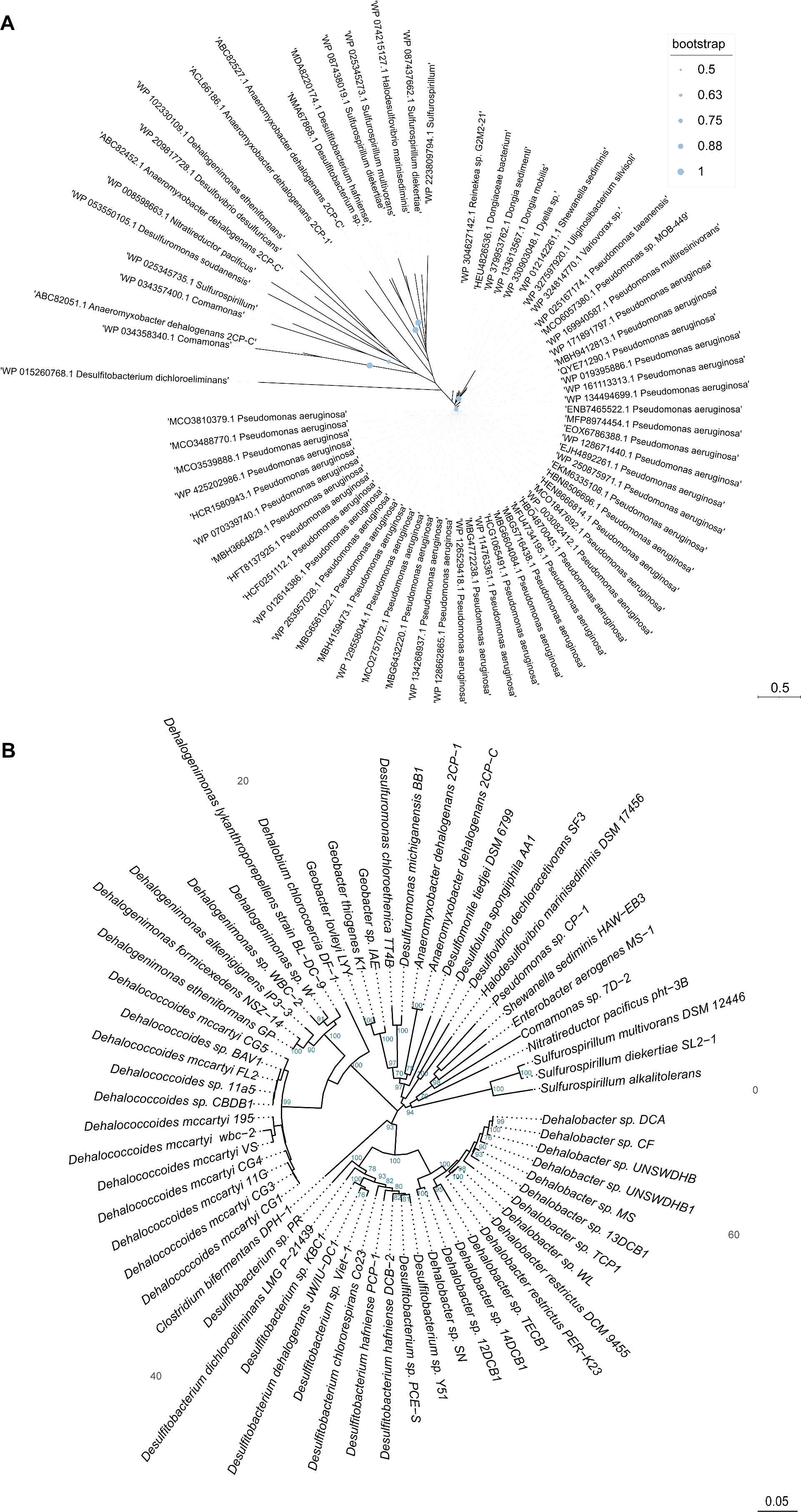


### **Figure S11.** **Diversity and phylogenetic distribution of EeuP homologs.** (**A**) Maximum likelihood phylogeny of EeuP homologs based on a Jones-Taylor-Thornoton (JTT) model with 1000 bootstrap replicates. Bootstrap support values greater than 50% are shown as blue circles. The scale bar indicates the number of amino acid substitutions per site. (**B**) Maximum likelihood phylogeny of 63 organohalide-respiring bacteria (OHRB) based on 16S rRNA gene sequences as reconstructed using the Kimura 2-parameter model with 1000 bootstrap replicates. Bootstrap values >70% are shown at corresponding nodes.


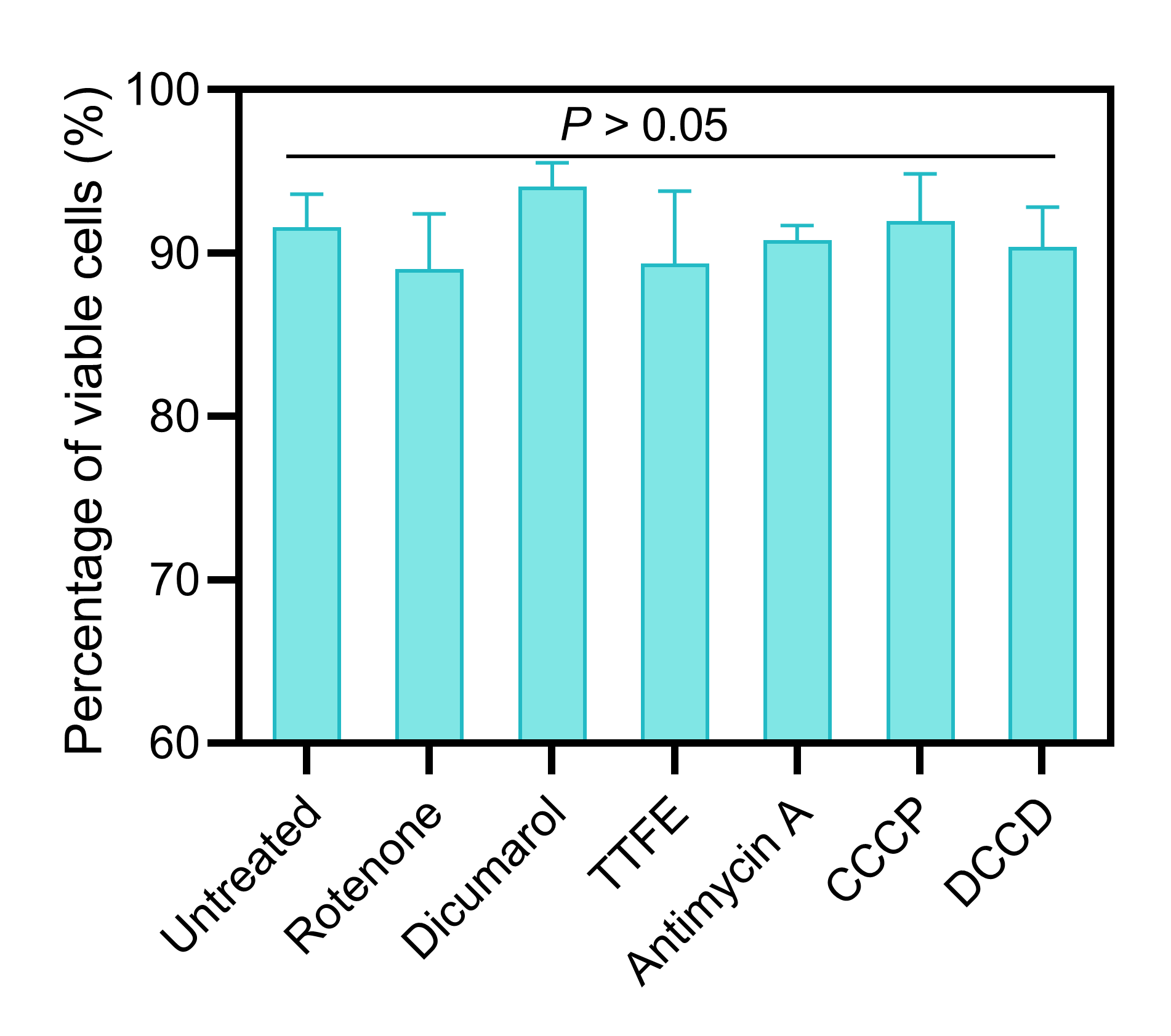


**Figure S12. Viability of strain CP-1 after treatment with various electron transport chain inhibitors.** Viability was assessed as the ratio of live to total cells, determined by LIVE/DEAD staining. Each test was conducted using 100 μM of the respective inhibitors (TTFE: Thenoyltrifluoroacetone; CCCP: Carbonyl cyanide m-chlorophenyl hydrazine; DCCD: Dicyclohexylcarbodiimide). Data are represented as means ± standard deviation from three biological replicates.


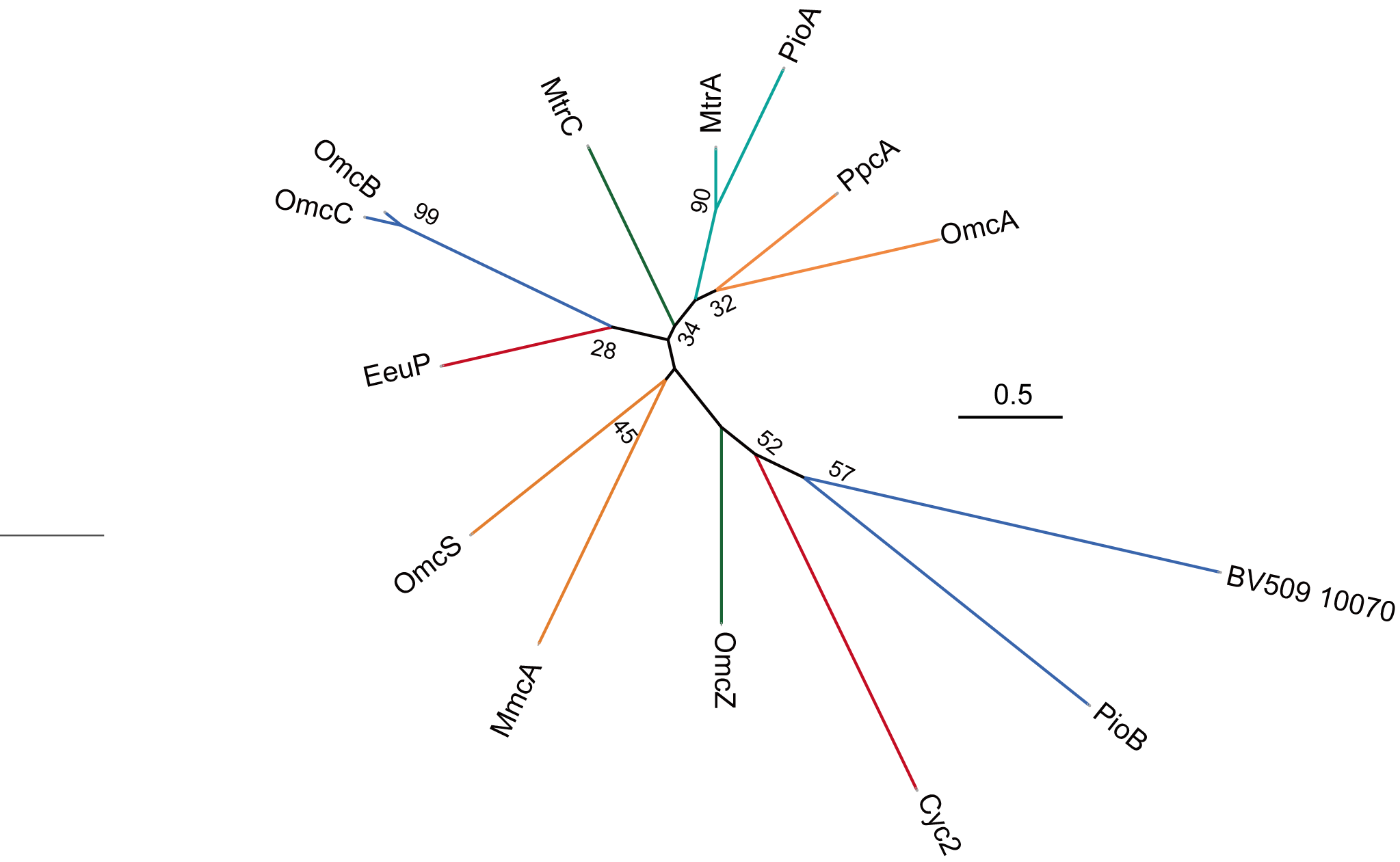


### **Figure S13. Phylogenetic analysis of EeuP and other reported porin-cytochromes.** Maximum-likelihood tree constructed from aligned amino acid sequences of redox-active cytochrome *c* proteins involved in extracellular electron transfer, including MtrA, MtrC, and OmcA from *Shewanella oneidensis* MR-1; PpcA, OmcS, OmcC, OmcB, and OmcZ from *Geobacter sulfurreducens* PCA; PioA and PioB from *Rhodopseudomonas palustris* TIE-1; BV509_10070 from *Rhodovulum sulfidophilum* AB26; Cyc2 from *Acidithiobacillus ferrooxidans*; and MmcA from *Methanosarcina acetivorans*. The tree was generated using MEGA12 with the Jones-Taylor-Thornoton (JTT) model and 1000 bootstrap replicates. Bootstrap values are indicated at nodes.


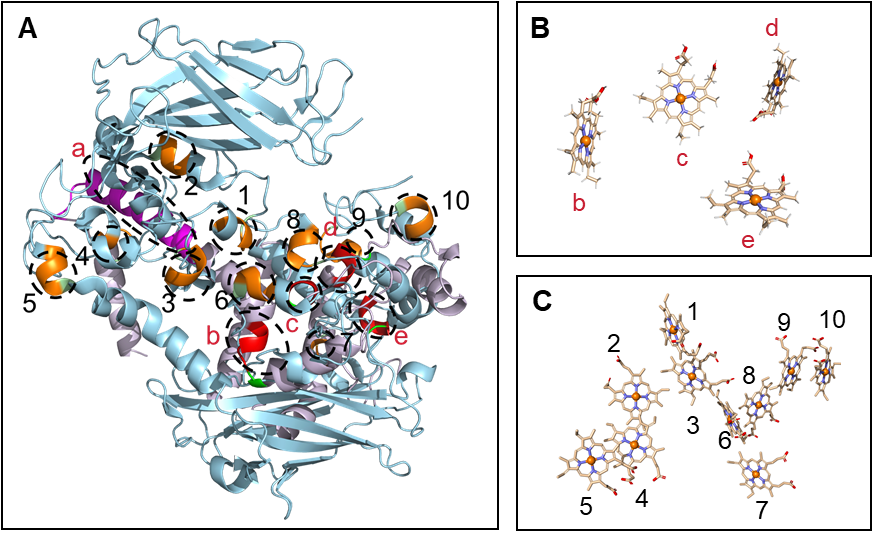


### **Figure S14. Comparative structure analysis of EeuP.** (**A**) Structural alignment of the AlphaFold3 predicted model of EeuP (198 aa, pink) with its closest available crystal structure, MtrC from *Shewenella oneidensis* MR-1 (PDB: 4LM8, cyan). The heme-binding motifs of MtrC (orange) and EeuP (red) are highlighted. The histidine (His) ligand of the ten bis-His coordinated heme groups (1–10) in MtrC (light green) and the four heme groups (b–e) in EeuP (dark green) are shown. The transmembrane helix of EeuP is indicated (purple, a). The arrangement of heme groups in the EeuP model (**B**) and in the MtrC crystal structure (**C**) is also presented. Structural alignment and model building were performed using the matchmaker tools in ChimeraX61.


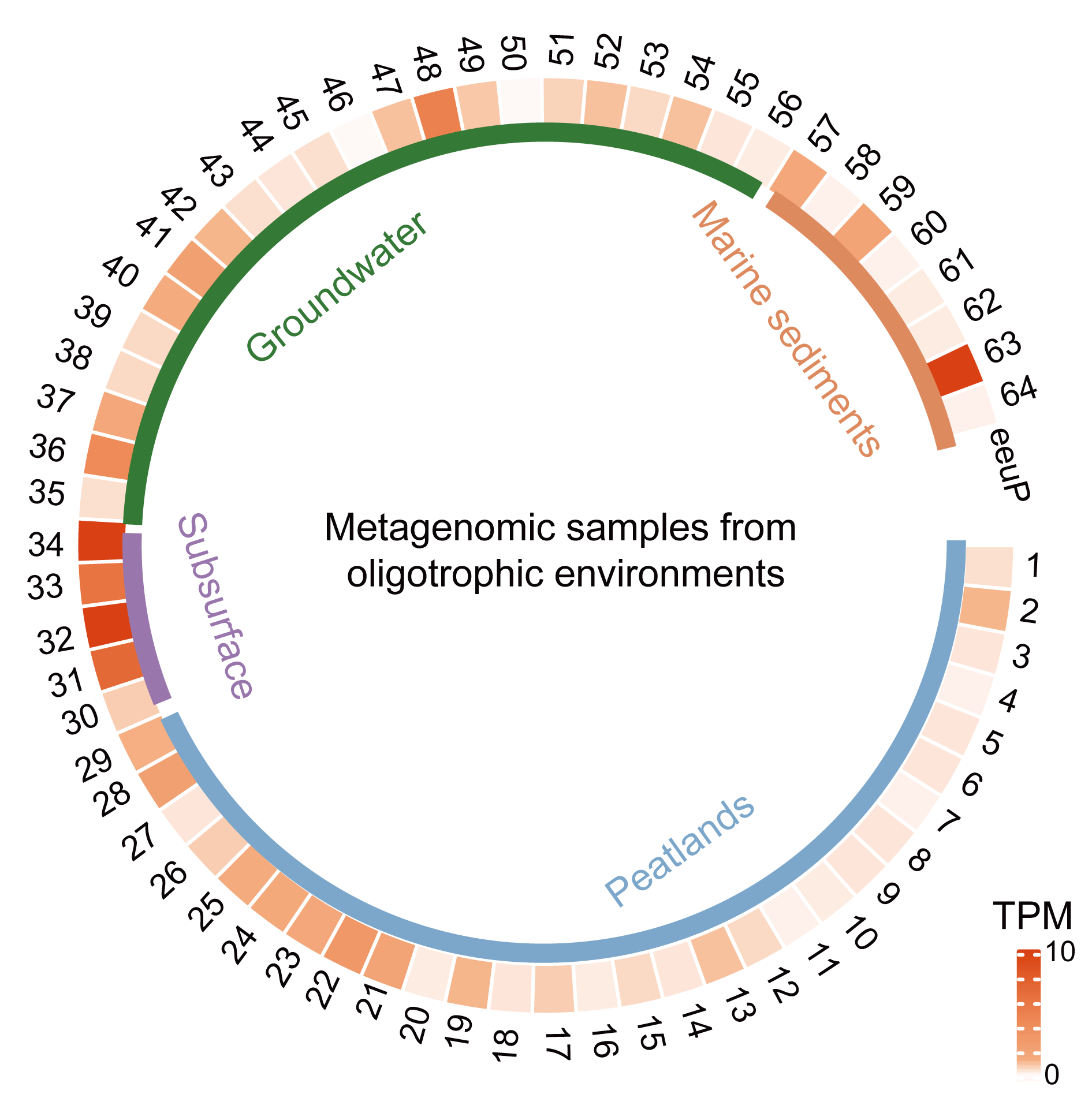


### **Figure S15. Distribution of EeuP homologs across 64 metagenomic datasets from oligotrophic environments.** Datasets were retrieved from NCBI SRA and ENA using SRA Toolkit (v3.2.1), with prefetch and fasterq-dump for FASTQ conversion. Included only Illumina-sequenced datasets with >100,000 reads and read lengths ≥150 bp. Raw reads were quality-filtered using Fastp (v0.36), host-derived sequences removed, and de novo assembly performed via a two-step strategy combining MEGAHIT and SPAdes, with intermediate Bowtie2 mapping. ORFs were predicted from contigs and translated into amino acid sequences using custom BioPython scripts. EeuP homologs were identified via a two-tier approach: (i) HMM search (e-value <1e^−5^; alignment coverage >50%) using profile-HMM (PF21342), and (ii) BLASTP validation against reference EeuP (e-value <1e^−5^; identity >30%; bit score >30). Gene abundance was quantified as transcripts per million (TPM), normalized by gene length and sample-wide read depth; per-metagenome abundance reflects summed TPM values for all identified EeuP homolog ORFs.


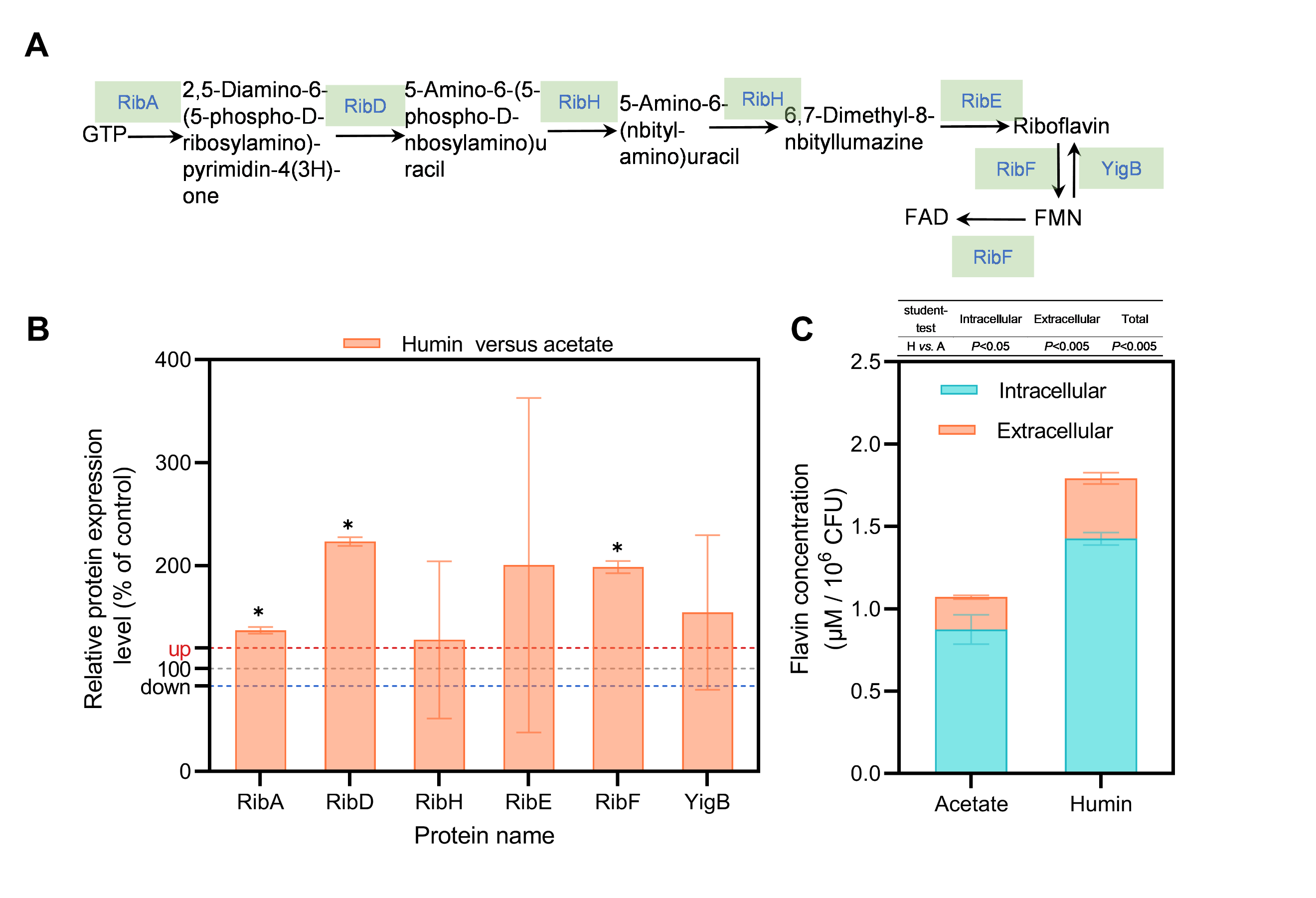


### **Figure S16. Biosynthesis and extracellular secretion of flavin-based electron mediators.** (**A**) Flavoprotein synthesis pathway; (**B**) expression levels of proteins involved in flavoprotein synthesis; (**C**) identification of intra- and extracellular flavins. Flavins were quantified by liquid chromatography-mass spectrometry. Data are represented as means ± standard deviation from three biologically independent replicates.


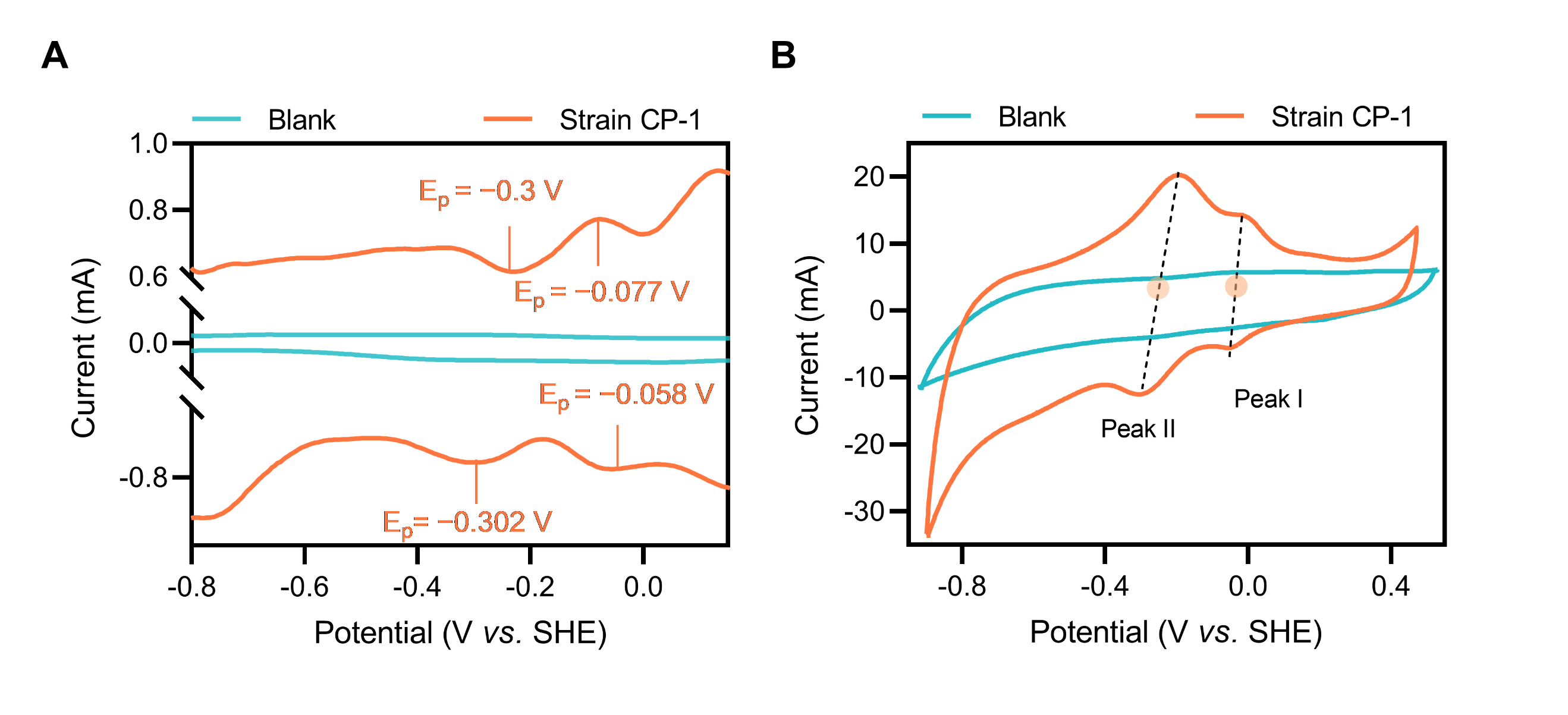


### **Figure S17.** **Electrochemical identification electron mediators in strain CP-1. (A**) Differential pulse voltammetry and (**B**) cyclic voltammetry of the strain CP-1 cathode biofilm.


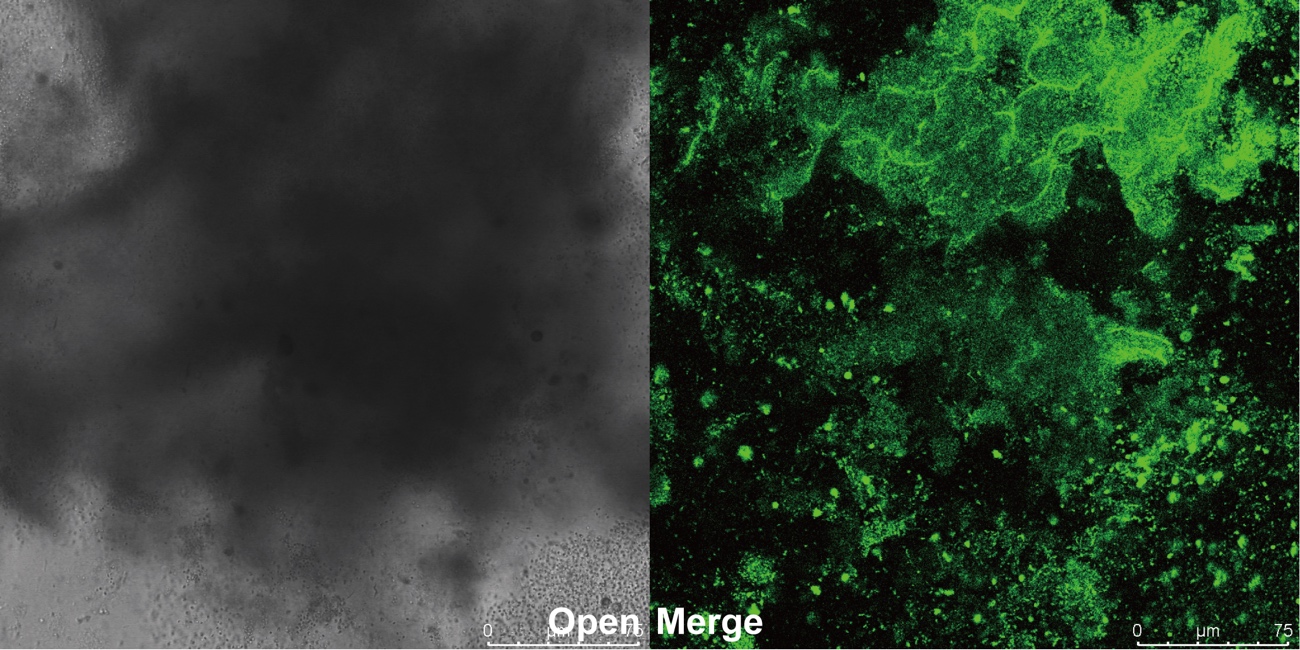


### **Figure S18**. **Confocal laser scanning microscopy of strain CP-1 on humin surfaces.** The left panel shows bright-field imaging, and cells were stained with DAPI. Scale bar: 75 μm.

### **Table S1.** Primers used for qPCR and mutant construction.

| ***Purpose*** | ***Primers*** | ***Sequence (5' to 3')*** | ***Amplicon Size (bp)*** |
| --- | --- | --- | --- |
| Quantitative PCR | *fixA*-F | TGGGCGTTTCCACCGCTTCCACCGT | 119 |
|  | *fixA*-R | CGCCTCGTTCTTCAGTTTCTCTACC |  |
|  | *fixB*-F | GCAGCTGTACATCGCGGTGGGCATC | 79 |
|  | *fixB*-R | CGCGACGATCACCTTCGAGTCCTTC |  |
|  | *cpr*A-F | ACGACCGCAGGAAGACCC | 225 |
|  | *cpr*A-R | GCCGCAGTTACCACAGATTTAC |  |
| Amplifying upstream homologous arm | *eeuP*-5F | GACATCCGCGACGCCGGCATCAC | 763 |
|  | *eeuP*-5R | GACATGACCGGGGTGAAGGGCTGGTAG |  |
| Amplifying downstream homologous arm | *eeuP*-3F | GATGAGTGCTTTCATGCGGCCTCCCTC | 815 |
|  | *eeuP*-3R | CTGTACAAGGCGGTGCCCGACGC |  |
| Amplification of gentamicin resistance gene | *eeuP*-GmF | CTACCAGCCCTTCACCCCGGTCATGTCttgtgacaatttaccgaacaactc | 943 |
|  | *eeuP*-GmR | AGGGAGGCCGCATGAAAGCACTCATCagaaatgcctcgacttcgc |  |
| Mutant verification primers | *eeuP*-outF | CTGCTGACCTCGCTGTCGACCCTGTC | 2644 |
|  | *eeuP*-outR | CAGGACTACCCGTTCTGGCTCGCCAC |  |
|  | *eeuP*-inF | GTGGCAGTCGATGCAGGTGGCTTC | 525 |
|  | *eeuP*-inR | CTGGCCGGTTACTGGCGGGTC |  |
| Quantifying *cprA* in field sample | Dehalo-F | TTATGGCGGTGCCAGTGT | 249 |
|  | Dehalo-R | GGGGTTCCTCCCACTATATTTT |  |

### **Table S2.** Growth and dechlorination rates for wild-type and ∆*eeu*P under different growth conditions ^a^.

| ***Strains*** | ***Aerobic cultivation*** | ***Anaerobic cultivation*** | |
| --- | --- | --- | --- |
|  | Specific growth rate (h^-1^) ^b^ | Specific growth rate (h^-1^) ^b^ | Dechlorination rate (h^-1^) |
| Wild-type | 0.132 ± 0.015 | 0.027 ± 0.004 | 0.024 ± 0.0050 |
| *∆eeuP* | 0.117 ± 0.019 | 0.024 ± 0.002 | 0.025 ± 0.0028 |

**Notes:**

**a.** For aerobic cultivation, strain CP-1 was grown in Luria-Bertani (LB) broth with oxygen as the electron acceptor (30°C, 150 rpm shaking).

**b.** Specific growth rates were determined by modified Gompertz model. No statistically significant difference between wild type and mutant (P > 0.05, t-test).

### **Table S3.** Effects of respiratory inhibitors on reductive dechlorination by strain CP-1 **^a^**.

| ***Inhibitors*** | ***TCP Removal Ratio (%)*** | | ***Dechlorination Rates (h^-1^)*** | | ***Electrons (mC)*** | |
| --- | --- | --- | --- | --- | --- | --- |
|  | ***Control*** | ***Treatment*** | ***Control*** | ***Treatment*** | ***Control*** | ***Treatment*** |
| Rotenone | 100.0±0.0 | 99.5±0.1 | 0.076±0.010 | 0.072±0.011 | 1150.32±84.42 | 1015.54±87.40 |
| Dicumarol |  | 100.0±0.0 |  | 0.055±0.008 |  | 730.91±62.25 |
| Thenoyltrifluoroacetone |  | 60.5±1.6 |  | 0.020±0.004 |  | 616.99±102.26 |
| Antimycin A |  | 66.1±10.0 |  | 0.019±0.004 |  | 497.71±107.45 |
| Carbonyl cyanide m-chlorophenyl hydrazine |  | 31.6±1.4 |  | 0.007±0.003 |  | 36.93±24.24 |
| Dicyclohexylcarbodiimide |  | 7.1±0.0 |  | 0.002±0.001 |  | 270.33±103.53 |

**Notes:**

**a.** Inhibitors were prepared as 10 mM stock solutions in anhydrous DMSO, stored at −20°C for ≤24 h prior to use, and added to the BES at a final concentration of 100 μM.

### **Table S4.** Metagenomic samples from oligotrophic peatlands, deep subsurface, groundwater, and marine environments.

| ***Habitats*** | ***SN*** | ***RUN*** | ***SRX*** |
| --- | --- | --- | --- |
| Peatlands | 1 | SRR5535863 | SRX2805480 |
|  | 2 | SRR5535864 | SRX2805479 |
|  | 3 | SRR5535865 | SRX2805478 |
|  | 4 | SRR5535866 | SRX2805477 |
|  | 5 | SRR5535867 | SRX2805476 |
|  | 6 | SRR5535868 | SRX2805475 |
|  | 7 | SRR5535869 | SRX2805474 |
|  | 8 | SRR5535870 | SRX2805473 |
|  | 9 | SRR5535871 | SRX2805472 |
|  | 10 | SRR5535872 | SRX2805471 |
|  | 11 | SRR5535877 | SRX2805466 |
|  | 12 | SRR5535881 | SRX2805462 |
|  | 13 | SRR5535882 | SRX2805461 |
|  | 14 | SRR5535883 | SRX2805460 |
|  | 15 | SRR5535884 | SRX2805459 |
|  | 16 | SRR5535885 | SRX2805458 |
|  | 17 | SRR5535886 | SRX2805457 |
|  | 18 | SRR5535887 | SRX2805456 |
|  | 19 | SRR5535888 | SRX2805455 |
|  | 20 | SRR5535889 | SRX2805454 |
|  | 21 | SRR31326370 | SRX26703900 |
|  | 22 | SRR31326371 | SRX26703899 |
|  | 23 | SRR31326372 | SRX26703898 |
|  | 24 | SRR31326373 | SRX26703897 |
|  | 25 | SRR31326374 | SRX26703896 |
|  | 26 | SRR31326375 | SRX26703895 |
|  | 27 | SRR31326376 | SRX26703894 |
|  | 28 | SRR31326377 | SRX26703893 |
|  | 29 | SRR31326378 | SRX26703892 |
| Deep subsurface | 30 | SRR5535890 | SRX2805453 |
|  | 31 | SRR8590643 | SRX5390933 |
|  | 32 | SRR8590644 | SRX5390932 |
|  | 33 | SRR8590645 | SRX5390931 |
|  | 34 | SRR8856338 | SRX5643996 |
| Groundwater | 35 | SRR30809809 | SRX26210515 |
|  | 36 | SRR30809810 | SRX26210514 |
|  | 37 | SRR30809811 | SRX26210513 |
|  | 38 | SRR30809812 | SRX26210512 |
|  | 39 | SRR30809813 | SRX26210511 |
|  | 40 | SRR30809814 | SRX26210510 |
|  | 41 | SRR30809815 | SRX26210509 |
|  | 42 | SRR30809816 | SRX26210508 |
|  | 43 | SRR30809817 | SRX26210507 |
|  | 44 | SRR30809818 | SRX26210506 |
|  | 45 | SRR30809819 | SRX26210505 |
|  | 46 | SRR30809820 | SRX26210504 |
|  | 47 | SRR30809821 | SRX26210503 |
|  | 48 | SRR15058747 | SRX11369042 |
|  | 49 | SRR8863439 | SRX5650853 |
|  | 50 | SRR8863440 | SRX5650852 |
|  | 51 | SRR8863431 | SRX5650849 |
|  | 52 | SRR8863432 | SRX5650848 |
|  | 53 | SRR8863433 | SRX5650847 |
|  | 54 | SRR8863434 | SRX5650846 |
|  | 55 | SRR8863435 | SRX5650845 |
|  | 56 | SRR8863436 | SRX5650844 |
| Marine sediments | 57 | SRR29965113 | SRX25446630 |
|  | 58 | SRR29936176 | SRX25429829 |
|  | 59 | SRR29936177 | SRX25429828 |
|  | 60 | SRR29965118 | SRX25446625 |
|  | 61 | SRR29965119 | SRX25446624 |
|  | 62 | SRR29965120 | SRX25446623 |
|  | 63 | SRR29965121 | SRX25446622 |
|  | 64 | SRR29965122 | SRX25446621 |

**Notes:**

**a.** Metagenomic datasets were downloaded from NCBI SRA and ENA.
